# Kalman Filtering for Attitude Estimation with Quaternions and Concepts from Manifold Theory

**DOI:** 10.3390/s19010149

**Published:** 2019-01-03

**Authors:** Pablo Bernal-Polo, Humberto Martínez-Barberá

**Affiliations:** Department of Information and Communication Engineering, University of Murcia, 30100 Murcia, Spain; humberto@um.es

**Keywords:** attitude, orientation, estimation, Kalman filter, quaternion, manifold

## Abstract

The problem of attitude estimation is broadly addressed using the Kalman filter formalism and unit quaternions to represent attitudes. This paper is also included in this framework, but introduces a new viewpoint from which the notions of “multiplicative update” and “covariance correction step” are conceived in a natural way. Concepts from manifold theory are used to define the moments of a distribution in a manifold. In particular, the mean and the covariance matrix of a distribution of unit quaternions are defined. Non-linear versions of the Kalman filter are developed applying these definitions. A simulation is designed to test the accuracy of the developed algorithms. The results of the simulation are analyzed and the best attitude estimator is selected according to the adopted performance metric.

## 1. Introduction

Mechanical state estimation of a vehicle is a field of interest. A vehicle is considered a rigid body, and its state of motion is represented by 4 mathematical objects: two of them represent its position and velocity, and the other two represent its orientation, and angular velocity. This paper is focused on the estimation of the angular state, composed of orientation, and angular velocity.

Although there are other mathematical tools used for estimation [1], the Kalman Filter [2] has become the algorithm par excellence in this area. Because of its simplicity, the rigor and elegance in its mathematical derivation, and its recursive nature it is very attractive for many practical applications. Its non-linear versions have been widely used in orientation estimation: the Extended Kalman Filter (EKF), and the Unscented Kalman Filter (UKF) [3]. However, there are problems arising from the used parametrization to represent the orientation.

The orientation of a system is represented by the rotation transformation that relates two reference frames: a reference frame anchored to that system, and an external reference frame. A thorough survey of attitude representations is provided in Reference [4]. The parametrization used to represent the rotation transformation could be singular, or present discontinuities among others. Table 1 summarizes the main characteristics of the most used parametrizations.

Having in mind that the *special orthogonal group* SO(3) has dimension three, ideally we would seek for a continuous and non-singular representation expressed by 3 parameters. However, since 1964 we know that “...it is topologically impossible to have a global 3-dimensional parametrization without singular points for the rotation group” [5]. Knowing this, we would not be wrong to say that unit quaternions are the most convenient representation we have, and that we will have for orientations. In Reference [6] the literature on attitude estimation is reviewed until 1982, when other parametrizations like Euler angles were common, and founds the basis of modern quaternion-based attitude estimation, in which this paper is supported. After that work, many others have explored this viewpoint, and have demonstrated its superiority [7,8,9,10,11,12].

Quaternions are 4-dimensional entities, but only those having unit norm represent a rotation transformation. This fact implies a problem in applying the ordinary Kalman Filter, so different approaches have emerged. Since a quaternion is of dimension 4, one tends to think at first on a 4×4 covariance matrix, and in the direct application of the Kalman Filter [13]. Given that all predictions are contained in the surface defined by the unit constraint, the covariance matrix shrinks in the orthogonal direction to this surface, which leads to a singular covariance matrix after several updates. A second perspective was firstly approached in Reference [6] and was after named as “Multiplicative Extended Kalman Filter” [8,11,12]. In this second approach we define an “error-quaternion” that is transformed to a 3-vector. We use this vector to build the covariance matrix, and we talk about a “3×3 representation of the quaternion covariance matrix”. However, there are still details in this adaptation that are currently being developed. Namely, the “covariance correction step” [14].

This paper presents a new viewpoint on the problem of attitude estimation using Kalman filters when the orientation is represented by unit quaternions. Noticing that unit quaternions live in a manifold (the unit sphere in $R^{4}$), we use basic concepts from manifold theory to define the mean and covariance matrix of a distribution of unit quaternions. With these definitions we develop two estimators based on the Kalman filter (one EKF-based and another UKF-based) arriving at the concepts of “multiplicative update” and “covariance correction step” in a natural and satisfying way. The inartificial emergence of these ideas establishes a solid foundation for the development of general navigation algorithms. Lastly, we also analyze the accuracy in the estimations of these two estimators using simulations.

The organization of this paper is as follows. In Section 2 we review quaternion basics. We also expose the new viewpoint on the definition of the quaternion mean and covariance matrix. In Section 3 we present the developed estimation algorithms. In Section 4 we define the performance metric, describe the simulation scheme, and present the results of the simulations. We also discuss the results. Finally, Section 5 concludes the paper.

## 2. Quaternions Describing Orientations

### 2.1. Quaternions

Quaternions are hypercomplex numbers composed of a real part and an imaginary part. The imaginary part is expressed using three different imaginary units {i,j,k} satisfying the Hamilton axiom:

(1) $$i^{2}=j^{2}=k^{2}=i*j*k=-1.$$

A quaternion q can be represented with 4 real numbers, and using several notations:

(2a) $$\begin{array}{cc} q & =q_{0}+q_{1}\;i+q_{2}\;j+q_{3}\;k\;\equiv \end{array}$$

(2b) $$\begin{array}{c} \equiv \;{\left(\;q_{0}\;,\;q_{1}\;,\;q_{2}\;,\;q_{3}\;\right)}^{T}\;\equiv \end{array}$$

(2c) $$\begin{array}{c} \equiv \;{\left(\;q_{0}\;,\;q\;\right)}^{T}. \end{array}$$

We will denote quaternions with bold italic symbols (q), while vectors will be denoted with bold upright symbols (q). Vectors will be written in matrix form, and the transposed of a matrix M will be denoted as $M^{T}$.

Quaternion product is defined by Equation (1) which produces the multiplication rule

(3) $$p*q=\left(\begin{array}{c} \;p_{0}\;q_{0}-p\cdot q\\ p_{0}\;q+q_{0}\;p+p\times q \end{array}\right),$$

where (·) represents the usual *dot product*, and (×) represents the 3-vector *cross product*. Note that the quaternion product (*) is different from the product denoted by (⊗) in reference [4]. Given this multiplication rule, the inverse of a quaternion q (the one for which $q*q^{-1}=q^{-1}*q=1$) is given by

(4) $$q^{-1}=\frac{\;1\;}{{\;∥q∥}^{2}\;}\;q^{*}=\frac{\;1\;}{{\;∥q∥}^{2}\;}\;{\left(\;q_{0}\;,\;-q\;\right)}^{T},$$

where $q^{*}$ represents the *complex conjugate quaternion*. Note that if q is a unit quaternion (a quaternion with ∥q∥=1), then $q^{-1}=q^{*}$.

### 2.2. Quaternions Representing Rotations

Each rotation transformation is mapped with a rotation matrix R and with two unit quaternions q and −q all of them related through

(5) $$R\left(q\right)=\left(\begin{array}{ccc} 1-2q_{2}^{2}-2q_{3}^{2} & 2\;(q_{1}\;q_{2}-q_{3}\;q_{0}) & 2\;(q_{1}\;q_{3}\;+\;q_{2}\;q_{0})\\ 2\;(q_{1}\;q_{2}\;+\;q_{3}\;q_{0}) & 1-2q_{1}^{2}-2q_{3}^{2} & 2\;(q_{2}\;q_{3}-q_{1}\;q_{0})\\ 2\;(q_{1}\;q_{3}-q_{2}\;q_{0}) & 2\;(q_{2}\;q_{3}\;+\;q_{1}\;q_{0}) & 1-2q_{1}^{2}-2q_{2}^{2} \end{array}\right).$$

Note that R(q)=R(−q).

Quaternions representing rotations have the form

(6) $$q={\left(\cos\left(\theta /2\right)\;\;,\;\;\hat{q}\;\sin\left(\theta /2\right)\;\;\right)}^{T},$$

where $\hat{q}$ denotes the unit vector that defines the rotation axis, and θ the angle of rotation. Having this form, they satisfy the restriction

(7) $${∥q∥}^{2}=q_{0}^{2}+q_{1}^{2}+q_{2}^{2}+q_{3}^{2}=1.$$

This means that quaternions describing rotations live in the unit sphere of $R^{4}$, $S^{3}$. This space is a manifold, and some concepts regarding these mathematical objects are useful in our context. In particular, the concept of chart is of special interest.

### 2.3. Distributions of Unit Quaternions

When dealing with the Kalman filter, the distribution of a random variable x is encoded by its mean $\bar{x}=E\left[x\right]$ and its covariance matrix P defined as

(8) $$P=E\left[\left(x-\bar{x}\right){\left(x-\bar{x}\right)}^{T}\right].$$

This definition makes sense when our random variables are defined in the Euclidean space. But how do we define the covariance matrix of a random variable living in a manifold like ours? How can we define the covariance for unit quaternions if $q-\bar{q}$ does not represent a rotation? (Unit quaternions form a group under multiplication, but not under addition. This means that the addition of two unit quaternions may not result in another unit quaternion. Therefore, the addition of two unit quaternions may not represent a rotation.) What would be the covariance matrix if each quaternion was equiprobable in the unit sphere? We cannot redefine the covariance matrix, because the Kalman filter uses this precise form in its derivations, but we can take advantage of the properties of a manifold. Let us retrieve some important definitions:

**Definition 1** **(Homeomorphism).** *A* homeomorphism *is a function f:X→Y between two topological spaces X and Y satisfying the next properties:*

- f is a bijection,
- f is continuous,
- its inverse function $f^{-1}$ is continuous.

*If such a function exists, we say that X and Y are * homeomorphic.

**Definition 2** **(Manifold).** *A n*-manifold *$M^{n}$ is a topological space in which each point is locally homeomorphic to the Euclidean space $R^{n}$. This is, each point $x\in M^{n}$ has a neighborhood $N\subset M^{n}$ for which we can define a homeomorphism $f:N\to B_{n}$ with $B_{n}$ the unit ball of $R^{n}$.*

**Definition 3** **(Chart).** *A* chart *for a manifold $M^{n}$ is a homeomorphism φ from an open subset $U\subset M^{n}$ to an open subset of the Euclidean space $V\subset R^{n}$. This is, a chart is a function*

(9) $$\varphi :U\subset M^{n}\to V\subset R^{n},$$

*with φ a homeomorphism. Traditionally, a chart is expressed as the pair (U,φ).*

Given these definitions we can continue our reasoning.

In Reference [8] it talks about four “attitude error representations”. Namely, the one we will call *Orthographic* (O), the *Rodrigues Parameters* (RP), the *Modified Rodrigues Parameters* (MRP), and the *Rotation Vector* (RV). The first three are what we know as *stereographic projections* (and are called *Orthographic*, *Gnomonic*, and *Stereographic* respectively). The last one is a projection called *Equidistant*. But all four are charts defining a homeomorphism from the manifold $S^{3}$ to the Euclidean space $R^{3}$. This is, they map a point q in the manifold with a point e in $R^{3}$. Table 2 arranges these chart definitions, together with their domain and image. We must ensure the charts to be bijections so that they properly define a homeomorphism, and that they do not map q and −q with different points of $R^{3}$ since they represent the same rotation. We achieve this by the given definitions of the domain and image for each chart.

**Figure 1:**
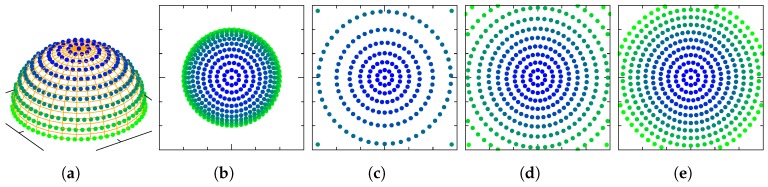
Points in the manifold with $q_{3}=0$ are mapped with points in the Euclidean space through each chart φ. (**a**) $S^{2}$; (**b**) O; (**c**) RP; (**d**) MRP; (**e**) RV.

**Figure 2:**
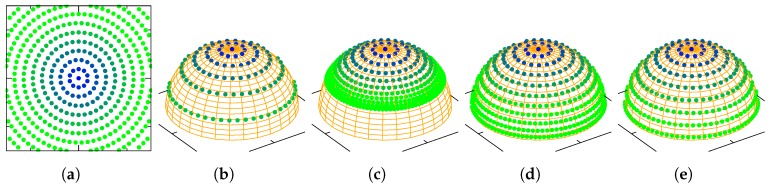
Points in the Euclidean space with $e_{3}=0$ are mapped with points in the manifold through each chart inverse $\varphi^{-1}$. (**a**) $R^{2}$; (**b**) O; (**c**) RP; (**d**) MRP; (**e**) RV.

Figure 1 shows how points in the sphere $S^{2}$ (subspace of the sphere $S^{3}$ where quaternions live) are mapped to points in $R^{2}$ (subspace of $R^{3}$ where the images of the charts are contained) through each one of the named charts. Since our charts are homeomorphisms, it is possible to invert the functions. Figure 2 shows how points from $R^{2}$ are mapped to points in the manifold through the inverted charts. As pointed in Reference [8], all four charts provide the same second-order approximation for a point $e\in R^{3}$ near the origin, to a quaternion $q\in S^{3}$

(10) $$\varphi^{-1}\left(e\right)\;\approx \;{\left(1\;-\;\frac{{\;∥e∥}^{2}\;}{\;8\;}\;\;,\;\;\frac{\;e\;}{\;2\;}\;\right)}^{T}.$$

We should notice that having $R^{3}$ and $S^{3}$ different metrics, a chart φ will inevitably produce a deformation of the space. However, for quaternions in the neighborhood of the identity quaternion (top of the sphere), our charts behave like the identity transformation between the imaginary part of these quaternions, and the points near the origin in $R^{3}$ as suggested by (10). This is a desirable property, as this means that the space around the identity quaternion closely resembles the Euclidean space, which is the space for which the Kalman filter is designed. But this just happens in the neighborhood of the identity quaternion. However, we can extend this property for any quaternion $\bar{q}\in S^{3}$ noting that any quaternion $q\in S^{3}$ can be expressed as a “deviation” from the first one through the quaternion product:

(11) $$q=\bar{q}*\delta^{\bar{q}},$$

where $\delta^{\bar{q}}$ represents such a deviation. (This definition is arbitrary: we could have chosen to relate the quaternions through $q=\delta^{\bar{q}}*\bar{q}$, but it is important to establish one of these definitions, and then be consequent with it. However, (11) entails a computational advantage for the computation of (37).) Then, we define a chart $\varphi_{\bar{q}}$ for each quaternion $\bar{q}\in S^{3}$ as

(12) $$e^{\bar{q}}=\varphi_{\bar{q}}\left(q\right)=\varphi \left(\;\delta^{\bar{q}}\;\right),$$

where $\delta^{\bar{q}}={\bar{q}}^{*}*q$ and where we have denoted the point of the Euclidean space mapped with the quaternion $q\in S^{3}$ through the chart $\varphi_{\bar{q}}$ as $e^{\bar{q}}$. Then, we will have a set of charts ${\left\{\varphi_{\bar{q}}\right\}}_{\bar{q}}$, each one resembling the Euclidean space around a quaternion $\bar{q}\in S^{3}$, and mapping this last quaternion to the origin of $R^{3}$. We will refer to the Euclidean space associated with the chart $\varphi_{\bar{q}}$ as the $\bar{q}$-centered chart. Thus, the homeomorphism $\varphi_{\bar{q}}^{-1}$ takes a point $e^{\bar{q}}$ in the $\bar{q}$-centered chart and maps it to a point q in the manifold through

(13) $$q=\varphi_{\bar{q}}^{-1}\left(\;e^{\bar{q}}\;\right)=\bar{q}*\varphi^{-1}\left(\;e^{\bar{q}}\;\right).$$

After reviewing these concepts, we can define the covariance matrix of a distribution of unit quaternions.

Given a unit quaternion $\bar{q}$ and a chart φ, we will define the expected value of a distribution of unit quaternions in the $\bar{q}$-centered chart as

(14) $${\bar{e}}^{\bar{q}}=E\left[\;e^{\bar{q}}\right],$$

and its covariance matrix as

(15) $$P^{\bar{q}}=E\left[\left(e^{\bar{q}}-{\bar{e}}^{\bar{q}}\right){\left(e^{\bar{q}}-{\bar{e}}^{\bar{q}}\right)}^{T}\right],$$

and the probability density of each unit quaternion q would be defined through the homeomorphism $q=\varphi_{\bar{q}}^{-1}\left(e^{\bar{q}}\right)$. Then, a distribution of unit quaternions needs of four mathematical objects to be encoded: $\left(\;\varphi \;,\;\bar{q}\;,\;{\bar{e}}^{\bar{q}}\;,\;P^{\bar{q}}\;\right)$. Although a distribution of unit quaternions is unique, given this definition, its expected value ${\bar{e}}^{\bar{q}}$ and its covariance matrix $P^{\bar{q}}$ may take different values depending on the chosen quaternion $\bar{q}$ and chart φ. However, knowing that the Kalman filter is designed for the Euclidean space, it will be convenient to choose a unit quaternion $\bar{q}$ central in the distribution, in order that the manifold space around it closely resembles the most significant region for the covariance matrix in the $\bar{q}$-centered chart. It is particularly convenient to choose a quaternion $\bar{q}$ such that ${\bar{e}}^{\bar{q}}=0$ so that the covariance matrix is centered in the origin of the $\bar{q}$-centered chart.

### 2.4. Transition Maps

At some step of the Kalman filter, we will have a distribution of unit quaternions defined in a $\bar{q}$-centered chart, and we will be interested in expressing our distribution in another $\bar{p}$-centered chart. The concept of transition map is relevant for this purpose.

**Definition 4** **(Transition map).** *Given two charts $\left(U_{\alpha },\varphi_{\alpha }\right)$ and $\left(U_{\beta },\varphi_{\beta }\right)$ for a manifold M, with $U_{\alpha \beta }=U_{\alpha }\cap U_{\beta }\neq \emptyset$, we can define a function $\varphi_{\alpha \beta }:\varphi_{\alpha }\left(\;U_{\alpha \beta }\;\right)\to \varphi_{\beta }\left(\;U_{\alpha \beta }\;\right)$ as*

(16) $$\varphi_{\alpha \beta }\left(x\right)\;=\;\varphi_{\beta }\left(\varphi_{\alpha }^{-1}\left(x\right)\right),$$

*with $x\in \varphi_{\alpha }\left(\;U_{\alpha \beta }\;\right)$. The function $\varphi_{\alpha \beta }$ is called a* transition map. *Being that $\varphi_{\alpha }$ and $\varphi_{\beta }$ are homeomorphisms, so is $\varphi_{\alpha \beta }$.*

For the present case, let us consider two unit quaternions $\bar{p}$ and $\bar{q}$ both related through

(17) $$\bar{p}=\bar{q}*\bar{\delta }.$$

These two quaternions define the charts $\varphi_{\bar{p}}$ and $\varphi_{\bar{q}}$. We build the transition map that relates a point $e^{\bar{q}}$ expressed in the $\bar{q}$-centered chart with a point $e^{\bar{p}}$ expressed in the $\bar{p}$-centered chart doing

(18a) $$\begin{array}{cc} e^{\bar{p}}\; & =\;\varphi_{\bar{p}}\left(\;\varphi_{\bar{q}}^{-1}\left(\;e^{\bar{q}}\;\right)\;\right)\;= \end{array}$$

(18b) $$\begin{array}{c} =\;\varphi \left(\;{\bar{p}}^{*}*\bar{q}*\varphi^{-1}\left(\;e^{\bar{q}}\;\right)\;\right)\;= \end{array}$$

(18c) $$\begin{array}{c} =\;\varphi \left(\;{\bar{\delta }}^{*}*\varphi^{-1}\left(\;e^{\bar{q}}\;\right)\;\right). \end{array}$$

That is to say, first we take the point $e^{\bar{q}}$ in the $\bar{q}$-centered chart, and we obtain its associated quaternion q in the manifold using $\varphi_{\bar{q}}^{-1}$. Then, we transform this quaternion q to a point $e^{\bar{p}}$ in the $\bar{p}$-centered chart. Nevertheless, knowing the quaternion $\bar{\delta }$ we do not need to explicitly compute q. In fact, being able to express the same quaternion q as two different deviations,

(19) $$\left.\begin{array}{c} q=\bar{q}*\delta^{\bar{q}}\\ q=\bar{p}*\delta^{\bar{p}} \end{array}\right\}\;\Rightarrow \;\delta^{\bar{p}}=\underset{{\bar{\delta }}^{*}}{\underset{︸}{{\bar{p}}^{*}*\bar{q}}}*\;\delta^{\bar{q}}.$$

Note the equivalence of expressions (18c) and (19).

**Figure 3:**
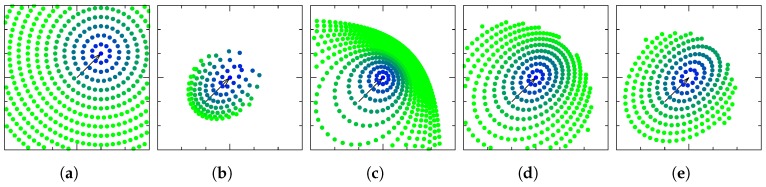
Points in a $\bar{q}$-centered chart are transformed using the transition map defined by each chart, and travel to the chart centered in the quaternion mapped with $e^{\bar{q}}={\left(1,1,0\right)}^{T}$ in the previous chart. (**a**) $R^{2}$; (**b**) O; (**c**) RP; (**d**) MRP; (**e**) RV.

Table 3 displays the transition maps for the charts studied. The detailed derivations of these transition maps can be found in Appendix A. Figure 3 attempts to provide some insight into how points are transformed through the transition map of each chart.

## 3. Manifold Kalman Filters

In this section we present the models adopted for the Manifold Kalman Filters (MKF), and we display the resulting algorithms.

The state of the system at a time *t* is defined by an orientation, encoded with a unit quaternion $q_{t}$ and by an angular velocity $\omega_{t}^{\prime }$. We will consider them to be random variables, and we will try to estimate their value using a Kalman filter.

Our unit quaternions $\left\{q_{t}\in H:∥q_{t}∥=1\right\}$ will define the rotation transformation that relates a vector $v_{t}^{\prime }$ expressed in a reference frame $S^{\prime }$ attached to the solid whose state we want to describe, with the same vector $v_{t}$ expressed in an external reference frame S

(20) $$v_{t}=R\left(q_{t}\right)\;v_{t}^{\prime }\;\equiv \;v_{t}=q_{t}*v_{t}^{\prime }*q_{t}^{*}.$$

For example, if we measure an acceleration $a_{t}^{\prime }$ in reference frame $S^{\prime }$ the acceleration in the inertial reference frame S would be given by $a_{t}=R\left(q_{t}\right)\;a_{t}^{\prime }$. This acceleration would be the one that we would have to integrate to obtain the position estimated by an accelerometer.

The vector $\omega_{t}^{\prime }$ will define the angular velocity of the solid measured in $S^{\prime }$. Note that we do not include the bias of the sensors in the state of our system. We will assume that our sensors are calibrated, so the biases are zero.

We can predict the value of the random variables that describe the state of our system through the following motion equations:

(21) $$\begin{array}{cc} \frac{\;d\;\omega^{\prime }\left(t\right)}{\;dt\;}\; & =\;q^{\omega }\left(t\right), \end{array}$$

(22) $$\begin{array}{cc} \frac{\;d\;q(t)\;}{\;dt\;}\; & =\;\frac{1}{2}\;q\left(t\right)*\omega^{\prime }\left(t\right)=\frac{1}{2}\;q\left(t\right)*\left(\begin{array}{c} \;0\;\;\\ \;\omega^{\prime }\left(t\right)\;\; \end{array}\right), \end{array}$$

where $q^{\omega }\left(t\right)$ is a random variable that represents the process noise, and is associated with the torque acting on the system, and with its inertia tensor. Its expected value at a given time *t* will be denoted as ${\bar{q}}_{t}^{\omega }$ and its covariance matrix will be denoted as $Q_{t}^{\omega }$.

We will assume that we have sensors giving measurements of angular velocity $\omega_{t}^{m}$ (which provide information about the relative change in orientation), and of a vector $v_{t}^{m}$ whose value $v_{t}$ expressed in the external reference frame S is known (this provides information about absolute orientation). Examples of such sensors could be a gyroscope giving angular velocity measurements, an accelerometer measuring the gravity vector near the Earth surface ($v_{t}:=-g$), or a magnetometer measuring the Earth magnetic field ($v_{t}:=B$). The measurement model relates these measurements with the variables that describe the state of the system:

(23) $$\begin{array}{cc} v_{t}^{m}\; & =\;R^{T}\left(q_{t}\right)\;\left(\;q_{t}^{v}+v_{t}\;\right)\;+\;r_{t}^{v}, \end{array}$$

(24) $$\begin{array}{cc} \omega_{t}^{m}\; & =\;\omega_{t}^{\prime }\;+\;r_{t}^{\omega }, \end{array}$$

where $r_{t}^{\omega }$ and $r_{t}^{v}$ are random variables with zero mean and covariance matrices $R_{t}^{\omega }$ and $R_{t}^{v}$ respectively that represent the measurement noises, and $q_{t}^{v}$ is another random variable with mean ${\bar{q}}_{t}^{v}$ and covariance matrix $Q_{t}^{v}$ representing external disturbances in the measurement of the vector $v_{t}$. For example, it could represent accelerations others than gravity for an accelerometer, or magnetic disturbances produced by moving irons for a magnetometer.

We will assume that the measurements arrive at discrete times ${\left\{t_{n}\right\}}_{n}$. The format $x_{t|t_{n}}$ will be used to denote a variable *x* at a time *t*, having included measurements up to a time $t_{n}$ with $t>t_{n}$. For the n-th time stamp, in which a measurement arrives, we will write $x_{t|n}$ for the sake of simplicity. Then, our knowledge about the state at a time *t*, having included measurements up to a time $t_{n}$ with $t>t_{n}$ is described by a distribution encoded in the collection of mathematical objects $\left(\;\varphi \;,\;\bar{p}\;,\;{\bar{x}}_{t|n}^{\bar{p}}\;,\;P_{t|n}^{\bar{p}}\;\right)$ as described in Section 2.3. For the present case, ${\bar{x}}_{t|n}^{\bar{p}}=\left(\;{\bar{e}}_{t|n}^{\bar{p}}\;,\;{\bar{\omega }}_{t|n}^{\prime }\;\right){}^{T}$ is the expected value of the distribution, and $P_{t|n}^{\bar{p}}$ is its 6×6 covariance matrix, both expressing the quaternion distribution in the $\bar{p}$-centered chart. Preferably, $\bar{p}$ will be a unit quaternion central in the distribution, so that the mapping of points from the $\bar{p}$-centered chart to the manifold causes minimal deformation in such distribution. The unit quaternion ${\bar{q}}_{t|n}=\varphi_{\bar{p}}^{-1}\left(\;{\bar{e}}_{t|n}^{\bar{p}}\;\right)$ will be our best estimation of the real quaternion $q_{t}$ that defines the orientation of the system with respect to the external reference frame S at time *t*.

The following subsections present the developed Kalman filters: one version based on the EKF and another version based on the UKF. The EKF is based on the linearization of the non-linear models to calculate the predicted covariance matrices. That is, the EKF approximates non-linear functions using their Jacobian matrices. To apply the EKF, our functions must be differentiable. On the other hand, the UKF is based on a deterministic sampling to approximate the distribution of our random variables. We select a minimal set of samples whose mean and covariance matrix are those of the state distribution. Then, they are transformed by the non-linear models, and the resulting set of points is used to compute the means and covariance matrices necessary to perform the Kalman update. This second approach does not need the functions to be differentiable.

### 3.1. Manifold Extended Kalman Filter

In this section we present the EKF-based estimator: the Manifold Extended Kalman Filter (MEKF). We offer here the main results of the more detailed derivation given in Appendix B.

A measurement

(25) $$z_{n}=\left(\begin{array}{c} \;v_{n}^{m}\\ \;\omega_{n}^{m} \end{array}\right)$$

arrives at time $t_{n}$. Our knowledge about the orientation at a previous time $t_{n-1}$ is described by a distribution expressed in the ${\bar{q}}_{n-1|n-1}$-centered chart. We assume that this distribution has mean

(26) $${\bar{x}}_{n-1|n-1}^{{\bar{q}}_{n-1|n-1}}=\left(\begin{array}{c} \;{\bar{e}}_{n-1|n-1}^{{\bar{q}}_{n-1|n-1}}=0\\ {\bar{\omega }}_{n-1|n-1}^{\prime }\;\; \end{array}\right),$$

and covariance matrix $P_{n-1|n-1}^{{\bar{q}}_{n-1|n-1}}$. This is, we have an initial four

(27) $$\left(\varphi ,\;{\bar{q}}_{n-1|n-1}\;,\;{\bar{\omega }}_{n-1|n-1}^{\prime }\;,\;P_{n-1|n-1}^{{\bar{q}}_{n-1|n-1}}\;\right).$$

The state prediction at time $t_{n}$ given all the information up to $t_{n-1}$ is computed through

(28) $$\begin{array}{cc} {\bar{\omega }}_{n|n-1}^{\prime }\; & =\;{\bar{\omega }}_{n-1|n-1}^{\prime }, \end{array}$$

(29) $$\begin{array}{cc} \delta_{n}^{\omega }\; & =\;\left(\begin{array}{c} \;\cos\left(\frac{\;∥{\bar{\omega }}_{n|n-1}^{\prime }∥\;\Delta t_{n}\;}{\;2\;}\right)\;\;\\ \;\frac{\;{\bar{\omega }}_{n|n-1}^{\prime }\;}{\;∥{\bar{\omega }}_{n|n-1}^{\prime }∥\;}\;\sin\left(\frac{\;∥{\bar{\omega }}_{n|n-1}^{\prime }∥\;\Delta t_{n}\;}{\;2\;}\right) \end{array}\right), \end{array}$$

(30) $$\begin{array}{cc} {\bar{q}}_{n|n-1}\; & =\;{\bar{q}}_{n-1|n-1}*\delta_{n}^{\omega }, \end{array}$$

(31) $$\begin{array}{cc} F_{n}\; & =\;\left(\begin{array}{cc} \;\;R^{T}\left(\delta_{n}^{\omega }\right)\; & \;I\;\Delta t_{n}\;\;\\ \;\;0\; & \;I\;\; \end{array}\right), \end{array}$$

(32) $$\begin{array}{cc} P_{n|n-1}^{{\bar{q}}_{n|n-1}}\; & =\;F_{n}\;\left[\;P_{n-1|n-1}^{{\bar{q}}_{n-1|n-1}}\;+\;Q_{n}\;\right]\;F_{n}^{T}, \end{array}$$

with

(33) $$Q_{n}=\left(\begin{array}{cc} \;\;Q_{n}^{\omega }\;\frac{{\left(\Delta t_{n}\right)}^{3}}{3}\; & \;-Q_{n}^{\omega }\;\frac{{\left(\Delta t_{n}\right)}^{2}}{2}\;\;\\ \;\;-Q_{n}^{\omega }\;\frac{{\left(\Delta t_{n}\right)}^{2}}{2}\; & \;Q_{n}^{\omega }\;\Delta t_{n}\;\; \end{array}\right).$$

The measurement prediction at the same time is given by

(34) $$\begin{array}{cc} {\bar{v}}_{n|n-1}^{m}\; & =\;R^{T}\left(\;{\bar{q}}_{n|n-1}\;\right)\;\left(\;{\bar{q}}_{n}^{v}+v_{n}\;\right), \end{array}$$

(35) $$\begin{array}{cc} {\bar{\omega }}_{n|n-1}^{m}\; & =\;{\bar{\omega }}_{n|n-1}^{\prime }, \end{array}$$

(36) $$\begin{array}{cc} {\bar{z}}_{n|n-1}\; & =\;\left(\begin{array}{c} \;{\bar{v}}_{n|n-1}^{m}\;\;\\ \;{\bar{\omega }}_{n|n-1}^{m}\;\; \end{array}\right), \end{array}$$

(37) $$\begin{array}{cc} H_{n}\; & =\;\left(\begin{array}{cc} \;\;{\left[\;{\bar{v}}_{n|n-1}^{m}\;\right]}_{\times }\; & 0\\ 0 & I \end{array}\right), \end{array}$$

(38) $$\begin{array}{cc} S_{n|n-1}\; & =\;H_{n}\;P_{n|n-1}^{{\bar{q}}_{n|n-1}}\;H_{n}^{T}\;+\;\left(\begin{array}{cc} \;\;R^{T}\left({\bar{q}}_{n|n-1}\right)\;Q_{n}^{v}\;R\left({\bar{q}}_{n|n-1}\right)+R_{n}^{v}\; & \;0\;\;\\ \;\;0\; & \;R_{n}^{\omega }\;\; \end{array}\right), \end{array}$$

where ${\left[v\right]}_{\times }$ stands for

(39) $${\left[v\right]}_{\times }=\left(\begin{array}{ccc} \;\;0 & -v_{3} & v_{2}\;\;\\ \;\;v_{3} & 0 & -v_{1}\;\;\\ \;\;-v_{2} & v_{1} & 0\;\; \end{array}\right).$$

At this point, we compute the Kalman gain $K_{n}$ and use it to obtain the optimal estimation of the state:

(40) $$\begin{array}{cc} K_{n}\; & =\;P_{n|n-1}^{{\bar{q}}_{n|n-1}}\;H_{n}^{T}\;S_{n|n-1}^{-1}, \end{array}$$

(41) $$\begin{array}{cc} {\bar{x}}_{n|n}^{{\bar{q}}_{n|n-1}}\; & =\;{\bar{x}}_{n|n-1}^{{\bar{q}}_{n|n-1}}\;+\;K_{n}\;\left(\;z_{n}\;-\;{\bar{z}}_{n|n-1}\;\right), \end{array}$$

(42) $$\begin{array}{cc} P_{n|n}^{{\bar{q}}_{n|n-1}}\; & =\;\left(\;I\;-\;K_{n}\;H_{n}\;\right)\;P_{n|n-1}^{{\bar{q}}_{n|n-1}}, \end{array}$$

where ${\bar{x}}_{n|n-1}^{{\bar{q}}_{n|n-1}}=\left(\;{\bar{e}}_{n|n-1}^{{\bar{q}}_{n|n-1}}=0\;,\;{\bar{\omega }}_{n|n-1}^{\prime }\;\right){}^{T}$. Finally, we need to obtain the updated unit quaternion, ${\bar{q}}_{n|n}$ and compute the mean and the covariance matrix in the ${\bar{q}}_{n|n}$-centered chart, so that the distribution is expressed in the same conditions as at the beginning of the iteration. The point ${\bar{e}}_{n|n}^{{\bar{q}}_{n|n-1}}$ that results from (41), and that is defined in the ${\bar{q}}_{n|n-1}$-centered chart, correspond to a unit quaternion in the manifold. This is the updated unit quaternion ${\bar{q}}_{n|n}$ which we are looking for:

(43a) $$\begin{array}{cc} {\bar{q}}_{n|n}\; & =\;\varphi_{{\bar{q}}_{n|n-1}}^{-1}\left(\;{\bar{e}}_{n|n}^{{\bar{q}}_{n|n-1}}\;\right)\;= \end{array}$$

(43b) $$\begin{array}{c} =\;{\bar{q}}_{n|n-1}*\varphi^{-1}\left(\;{\bar{e}}_{n|n}^{{\bar{q}}_{n|n-1}}\;\right)\;= \end{array}$$

(43c) $$\begin{array}{c} =\;{\bar{q}}_{n|n-1}*{\bar{\delta }}_{n}. \end{array}$$

Knowing that the Kalman update (41) could produce any point in the ${\bar{q}}_{n|n-1}$-centered chart we will need to “saturate” to the closest point contained in the image of each chart. The point ${\bar{e}}_{n|n}^{{\bar{q}}_{n|n-1}}$ in the ${\bar{q}}_{n|n-1}$-centered chart is the origin in the ${\bar{q}}_{n|n}$-centered chart. Then, the expected value of the state in this new chart will be given by ${\bar{x}}_{n|n}^{{\bar{q}}_{n|n}}=\left(\;{\bar{e}}_{n|n}^{{\bar{q}}_{n|n}}=0\;,\;{\bar{\omega }}_{n|n}^{\prime }\;\right){}^{T}$ as at the beginning of the iteration.

To update the covariance matrix we need to consider its definition (15). We want to compute $P^{{\bar{q}}_{n|n}}$ having $P^{{\bar{q}}_{n|n-1}}$ and knowing the relation $e^{\bar{p}}\left(\;e^{\bar{q}}\;\right)$ provided by the transition maps in Table 3. Continuing with the EKF philosophy, the update for the covariance matrix will be found by linearizing $e^{\bar{p}}\left(\;e^{\bar{q}}\;\right)$ around the point where the majority of information is comprised (in our case, the point ${\bar{e}}^{\bar{q}}={\bar{e}}_{n|n}^{{\bar{q}}_{n|n-1}}$):

(44) $$\begin{array}{cc} e_{i}^{\bar{p}}\left(\;e^{\bar{q}}\;\right)\; & =\;e_{i}^{\bar{p}}\left(\;{\bar{e}}^{\bar{q}}\;\right)\;\;+\;\;\sum_{j}{\left.\frac{\;\partial \;e_{i}^{\bar{p}}\left(\;e^{\bar{q}}\;\right)\;}{\;\partial e_{j}^{\bar{q}}\;}\right|}_{e^{\bar{q}}={\bar{e}}^{\bar{q}}}\;\;\;\left(\;e_{j}^{\bar{q}}-{\bar{e}}_{j}^{\bar{q}}\;\right)+O\left(∥e^{\bar{q}}-{\bar{e}}^{\bar{q}}{∥}^{2}\right), \end{array}$$

where we have used the *big O notation* to describe the limiting behavior of the error term of the approximation as $e^{\bar{q}}\to {\bar{e}}^{\bar{q}}$. In particular, if we define

(45) $${\left(T\right)}_{ij}={\left.\frac{\;\partial \;e_{i}^{\bar{p}}\left(\;e^{\bar{q}}\;\right)\;}{\;\partial e_{j}^{\bar{q}}\;}\right|}_{e^{\bar{q}}={\bar{e}}^{\bar{q}}},$$

then,

(46) $$e^{\bar{p}}-{\bar{e}}^{\bar{p}}\;\approx \;e^{\bar{p}}\left(\;e^{\bar{q}}\;\right)\;-\;e^{\bar{p}}\left(\;{\bar{e}}^{\bar{q}}\;\right)\;\approx \;T\;\left(e^{\bar{q}}-{\bar{e}}^{\bar{q}}\right),$$

and the final update for the covariance matrix will be computed through

(47a) $$\begin{array}{cc} P_{n|n}^{{\bar{q}}_{n|n}}\; & =\;E\left[\;\left(x_{n|n}^{{\bar{q}}_{n|n}}-{\bar{x}}_{n|n}^{{\bar{q}}_{n|n}}\right)\;{\left(x_{n|n}^{{\bar{q}}_{n|n}}-{\bar{x}}_{n|n}^{{\bar{q}}_{n|n}}\right)}^{T}\;\right]\;\approx \end{array}$$

(47b) $$\begin{array}{c} \approx \;\left(\begin{array}{cc} \;\;T(\;{\bar{\delta }}_{n}\;)\; & \;0\\ \;\;0\; & \;I \end{array}\right)\;P_{n|n}^{{\bar{q}}_{n|n-1}}\;{\left(\begin{array}{cc} \;\;T(\;{\bar{\delta }}_{n}\;)\; & \;0\\ \;\;0\; & \;I \end{array}\right)}^{T}. \end{array}$$

Table 4 summarizes the resulting T-matrix for each chart, along with their application domain. A detailed derivation of these T-matrices can be found in Appendix C.

After the final computation we obtain the four

(48) $$\left(\varphi ,\;{\bar{q}}_{n|n}\;,\;{\bar{\omega }}_{n|n}^{\prime }\;,\;P_{n|n}^{{\bar{q}}_{n|n}}\;\right),$$

that is a condition equivalent to (27) in which we started the iteration.

### 3.2. Manifold Unscented Kalman Filter

In this section we present the UKF-based estimator: the Manifold Unscented Kalman Filter (MUKF).

A measurement $z_{n}$ arrives at time $t_{n}$. Our knowledge about the orientation at a previous time $t_{n-1}$ is described by a distribution expressed in the ${\bar{q}}_{n-1|n-2}$-centered chart. This distribution is encoded in the four

(49) $$\left(\;\varphi \;\;,\;\;{\bar{q}}_{n-1|n-2}\;\;,\;\;{\bar{x}}_{n-1|n-1}^{{\bar{q}}_{n-1|n-2}}\;\;,\;\;P_{n-1|n-1}^{{\bar{q}}_{n-1|n-2}}\;\right)\;.$$

The first step in the UKF is to create the augmented N×1 mean ${\tilde{x}}_{n}$ and N×N covariance matrix ${\tilde{P}}_{n}$. Since the measurement equations are linear for the random variables $r_{t}^{\omega }$ and $r_{t}^{v}$ we can leave their covariance matrices out of the augmented one and add them later:

(50) $$\begin{array}{cc} {\tilde{x}}_{n}\; & =\;\left(\begin{array}{c} \;{\bar{x}}_{n-1|n-1}^{{\bar{q}}_{n-1|n-2}}\;\;\\ \;{\bar{q}}_{n}^{\omega }\;\;\\ \;{\bar{q}}_{n}^{v}\;\; \end{array}\right), \end{array}$$

(51) $$\begin{array}{cc} {\tilde{P}}_{n}\; & =\;\left(\begin{array}{ccc} P_{n-1|n-1}^{{\bar{q}}_{n-1|n-2}} & 0 & 0\\ 0 & Q_{n}^{\omega } & 0\\ 0 & 0 & Q_{n}^{v} \end{array}\right). \end{array}$$

Then, we obtain the matrix $L_{n}$ which satisfies $L_{n}\;L_{n}^{T}={\tilde{P}}_{n}$ and we use it to generate the 2N+1 sigma points ${\left\{X_{j}\right\}}_{j=0}^{2N}$ as described in Ref. [15]:

(52a) $$\begin{array}{cc} X_{i,0}\; & =\;{\left({\tilde{x}}_{n}\right)}_{i}, \end{array}$$

(52b) $$\begin{array}{cc} X_{i,j}\; & =\;{\left({\tilde{x}}_{n}\right)}_{i}\;+\;\frac{\;{\left(L_{n}\right)}_{ij}\;}{\;\sqrt{2W_{j}\;}\;}\;\mathrm{for}\;j=1,\;\ldots \;,N, \end{array}$$

(52c) $$\begin{array}{cc} X_{i,j+N}\; & =\;{\left({\tilde{x}}_{n}\right)}_{i}\;-\;\frac{\;{\left(L_{n}\right)}_{ij}\;}{\;\sqrt{2W_{j}\;}\;}\;\mathrm{for}\;j=1,\;\ldots \;,N, \end{array}$$

being $W_{j}=\left(1-W_{0}\right)/\left(2N\right)$ for j≠0 where $W_{0}$ regulates the importance given to the sigma point $X_{0}$ in the computation of the mean. These sigma points ${\left\{X_{j}\right\}}_{j}$ are expressed in the ${\bar{q}}_{n-1|n-2}$-centered chart. We need to express them in the manifold before applying the evolution equations and the measurement equations:

(53) $$\begin{array}{cc} X_{j}^{q}\; & =\;\varphi_{{\bar{q}}_{n-1|n-2}}^{-1}\left(\;X_{j}^{e}\;\right)\;=\;{\bar{q}}_{n-1|n-2}*\varphi^{-1}\left(\;X_{j}^{e}\;\right), \end{array}$$

(54) $$\begin{array}{cc} Y_{j}^{\omega }\; & =\;X_{j}^{\omega }\;\;+\;\;X_{j}^{q^{\omega }}\;\Delta t_{n}, \end{array}$$

(55) $$\begin{array}{cc} Y_{j}^{q}\; & =\;X_{j}^{q}*\left(\begin{array}{c} \;\cos\left(\frac{\;∥Y_{j}^{\omega }∥\;\Delta t_{n}\;}{\;2\;}\right)\;\;\\ \;{\hat{Y}}_{j}^{\omega }\;\sin\left(\frac{\;∥Y_{j}^{\omega }∥\;\Delta t_{n}\;}{\;2\;}\right)\;\; \end{array}\right), \end{array}$$

(56) $$\begin{array}{cc} Z_{j}^{v}\; & =\;R^{T}\left(X_{j}^{q}\right)\;\left(\;X_{j}^{v}+v_{t}\;\right), \end{array}$$

(57) $$\begin{array}{cc} Z_{j}^{\omega }\; & =\;Y_{j}^{\omega }, \end{array}$$

where for the *j*-th sigma point, $X_{j}^{e}$ is its chart point part and $X_{j}^{q}$ is the quaternion with which it is mapped, $X_{j}^{\omega }$ is its angular velocity part, $X_{j}^{q^{\omega }}$ is its angular velocity noise part, $Y_{j}^{\omega }$ is its angular velocity prediction, $Y_{j}^{q}$ is the quaternion part of its prediction (we have assumed that the angular velocity $Y_{j}^{\omega }$ is constant in the time interval $\left[t_{n-1},t_{n}\right)$ so that we can use (A20)), $X_{j}^{v}$ is the vector process noise part, $Z_{j}^{v}$ is its vector measurement prediction, $Z_{j}^{\omega }$ is its angular velocity measurement prediction, and $\Delta t_{n}=t_{n}-t_{n-1}$. Note that when applying the inverse chart $\varphi^{-1}$ we will need to “saturate” $X_{j}^{e}$ to the closest point in the image of φ. Having these new sigma points, we can obtain the means and covariance matrices of the distributions present in the UKF. First, defining $Z_{j}:=\left(\;Z_{j}^{v}\;,\;Z_{j}^{\omega }\;\right){\;}^{T}$ the means are computed through

(58) $$\begin{array}{cc} {\bar{q}}_{n|n-1}\; & =\;\frac{\;\sum_{j}\;W_{j}\;Y_{j}^{q}\;}{\;∥\sum_{j}\;W_{j}\;Y_{j}^{q}∥\;}, \end{array}$$

(59) $$\begin{array}{cc} {\bar{\omega }}_{n|n-1}^{\prime }\; & =\;\sum_{j}\;W_{j}\;Y_{j}^{\omega }, \end{array}$$

(60) $$\begin{array}{cc} {\bar{x}}_{n|n-1}^{{\bar{q}}_{n|n-1}}\; & =\;\left(\begin{array}{c} \;\varphi_{{\bar{q}}_{n|n-1}}\left({\bar{q}}_{n|n-1}\right)=0\;\;\\ \;{\bar{\omega }}_{n|n-1}^{\prime }\;\; \end{array}\right), \end{array}$$

(61) $$\begin{array}{cc} {\bar{z}}_{n|n-1}\; & =\;\sum_{j}W_{j}\;Z_{j}. \end{array}$$

where we have used a variation of the result provided in Ref. [16]. Namely,

(62) $$\bar{q}\;\approx \;\frac{\sum_{j}q_{j}}{∥\sum_{j}q_{j}∥},$$

with $q_{j}\cdot q_{k}>0$ for j,k=0,…,2N. This result is shown to minimize the fourth order approximation of the distance defined as the sum of squared angles between the rotation transformation represented by each quaternion $q_{j}$ and the one represented by $\bar{q}$. This approach to compute the mean quaternion is extremely efficient, and its derivation is elegant and simple. In order to ensure that $q_{j}\cdot q_{k}>0$ it is useful to remember the property that both q and −q represent the same rotation. This property is also useful for introducing the quaternions in the domain of φ to execute the next step of the filter.

After this, we use the obtained mean quaternion ${\bar{q}}_{n|n-1}$ to express each sigma point in the ${\bar{q}}_{n|n-1}$-centered chart, and compute the covariance matrices:

(63) $$\begin{array}{cc} Y_{j}^{e}\; & =\;\varphi_{{\bar{q}}_{n|n-1}}\left(\;Y_{j}^{q}\;\right)\;=\;\varphi \left(\;{\bar{q}}_{n|n-1}^{*}*Y_{j}^{q}\;\right), \end{array}$$

(64) $$\begin{array}{cc} P_{n|n-1}^{{\bar{q}}_{n|n-1}}\; & =\;\sum_{j}\;W_{j}\;Y_{j}\;Y_{j}^{T}, \end{array}$$

(65) $$\begin{array}{cc} P_{n|n-1}^{yz}\; & =\;\sum_{j}\;W_{j}\;Y_{j}{\left(Z_{j}-{\bar{z}}_{n|n-1}\right)}^{T}, \end{array}$$

(66) $$\begin{array}{cc} S_{n|n-1}\; & =\;\sum_{j}\;W_{j}\;\left(Z_{j}-{\bar{z}}_{n|n-1}\right){\left(Z_{j}-{\bar{z}}_{n|n-1}\right)}^{T}\;\;+\;\;\left(\begin{array}{cc} \;\;R_{n}^{v}\; & \;0\;\;\\ \;\;0\; & \;R_{n}^{\omega }\;\; \end{array}\right), \end{array}$$

where we have denoted $Y_{j}:=\left(\;Y_{j}^{e}\;,\;Y_{j}^{\omega }-{\bar{\omega }}_{n|n-1}^{\prime }\;\right){}^{T}$. Finally, we compute the UKF version of the Kalman gain $K_{n}$ and we use it to obtain the optimal estimation of the state:

(67) $$\begin{array}{cc} K_{n}\; & =\;P_{n|n-1}^{yz}\;S_{n|n-1}^{-1}, \end{array}$$

(68) $$\begin{array}{cc} {\bar{x}}_{n|n}^{{\bar{q}}_{n|n-1}}\; & =\;{\bar{x}}_{n|n-1}^{{\bar{q}}_{n|n-1}}\;\;+\;\;K_{n}\;\left(\;z_{n}\;-\;{\bar{z}}_{n|n-1}\;\right), \end{array}$$

(69) $$\begin{array}{cc} P_{n|n}^{{\bar{q}}_{n|n-1}}\; & =\;P_{n|n-1}^{{\bar{q}}_{n|n-1}}\;\;-\;\;K_{n}\;S_{n|n-1}\;K_{n}^{T}, \end{array}$$

arriving at the same conditions in which we began the iteration, with a distribution expressed in the ${\bar{q}}_{n|n-1}$-centered chart, and encoded by the four

(70) $$\left(\;\varphi \;,\;{\bar{q}}_{n|n-1}\;,\;{\bar{x}}_{n|n}^{{\bar{q}}_{n|n-1}}\;,\;P_{n|n}^{{\bar{q}}_{n|n-1}}\;\right).$$

Our best estimation for the orientation at this time is

(71) $${\bar{q}}_{n|n}=\varphi_{{\bar{q}}_{n|n-1}}^{-1}\left(\;{\bar{e}}_{n|n}^{{\bar{q}}_{n|n-1}}\;\right)={\bar{q}}_{n|n-1}*\varphi^{-1}\left(\;{\bar{e}}_{n|n}^{{\bar{q}}_{n|n-1}}\;\right),$$

being ${\bar{e}}_{n|n}^{{\bar{q}}_{n|n-1}}$ the part of the mean ${\bar{x}}_{n|n}^{{\bar{q}}_{n|n-1}}$ that represent the quaternion in the ${\bar{q}}_{n|n-1}$-centered chart.

Note that setting ${\bar{q}}_{n-1|n-2}:={\bar{q}}_{n-1|n-1}$ and ${\bar{e}}_{n-1|n-1}^{{\bar{q}}_{n-1|n-2}}:=0$ at the beginning of each iteration yields the traditional version of the algorithm, where a “reset operation” is performed instead of the covariance matrix update.

## 4. Simulation Results

This section presents the results of the simulations used to measure the accuracy of each estimator. Simulations are chosen instead of real experiments because a real system entails an uncertainty in the measurement of the true attitude: the attitude that is used to compare with that estimated by the algorithms. There are sources of error ranging from a miscalibration of the measurement system to a possible bias in the “true attitude” produced by another attitude estimator, which makes it problematic to define an adequate metric to measure the accuracy of the algorithms. For this reason, the authors consider that using a simulation is more reliable to avoid possible biases in the results due to said sources of error. Others have performed similar types of tests [7,17]. However, the results do not seem to be statistically conclusive: only the estimations of some orientation trajectories are shown.

We perform our comparison through a simulation in which we do have an absolute knowledge of the attitude of the system: a true oracle exists in a simulation. Therefore, we can compare the real orientation with the attitude estimated by the algorithms having fed them only with simulated measurements that we obtain from such known orientations. We will extract our performance metrics from a wide set of orientation trajectories in order to obtain statistically conclusive results.

We try to answer three questions with the simulation test. The first question is, is there a chart for which we get a greater accuracy in attitude estimation? The second one is, what algorithm produces the most accurate attitude estimation, the MEKF or the MUKF? The last question stems from the fact that previous algorithms on attitude estimation, such as the Multiplicative Extended Kalman Filter, did not contemplate updating the distribution from one chart to another as done at (47b) in the MEKF. However, their estimators performed well [6,7,12]. Then the third question is, does this “chart update” imply an improvement in the accuracy of the attitude estimation?

Although a simulation has been used to compare our algorithms, these have also been tested with a real IMU. In the Supplementary Materials one can find a demonstration video, the source code used in the video, the source code used to generate the simulations, and the source code used to obtain the computational cost of the algorithms in each platform.

### 4.1. Performance Metric

We have already described a quaternion q as a deviation from another quaternion $\bar{q}$ as $q=\bar{q}*\delta$. Now we define the instantaneous error between an estimated attitude, represented by a unit quaternion $\bar{q}$ and the real attitude, represented by the unit quaternion $\overset{⭑}{q}$ as the angle we have to rotate one of them to transform it into the other. This is, the angle of the rotation transformation defined by the quaternion $\delta_{e}$ such that $\overset{⭑}{q}=\bar{q}*\delta_{e}$. Recalling (6), this angle can be computed as:

(72a) $$\begin{array}{cc} \theta_{e}\; & =\;2\;\arccos\left[\;{\left({\bar{q}}^{*}*\overset{⭑}{q}\right)}_{0}\;\right]\;= \end{array}$$

(72b) $$\begin{array}{c} =\;2\;\arccos\left(\;\bar{q}\cdot \overset{⭑}{q}\;\right), \end{array}$$

having previously ensured that $\bar{q}\cdot \overset{⭑}{q}\geq 0$ using the fact that both q and −q represent the same rotation transformation.

Angle $\theta_{e}$ will vary along an orientation trajectory. Then, we will define the mean error in orientation estimation for a given trajectory starting at time t=0 and ending at time t=T as

(73) $$e_{\theta }=\frac{1}{T}\int_{0}^{T}\theta_{e}\left(t\right)\;dt.$$

Finally, $e_{\theta }$ will depend on the followed trajectory, and on the set of taken measurements. We will need to generate several orientation trajectories to obtain the mean value ${\bar{e}}_{\theta }$ and the variance $\sigma_{{\bar{e}}_{\theta }}^{2}$ that characterize the distribution of the error in orientation estimation $e_{\theta }$ for each algorithm. We will define the confidence interval for the computed ${\bar{e}}_{\theta }$ as

(74) $$\left[\;{\bar{e}}_{\theta }-3\;\sigma_{{\bar{e}}_{\theta }}/\sqrt{N_{s}}\;,\;{\bar{e}}_{\theta }+3\;\sigma_{{\bar{e}}_{\theta }}/\sqrt{N_{s}}\;\right],$$

where $N_{s}$ is the number of samples taken for the ${\bar{e}}_{\theta }$ computation, so that $\sigma_{{\bar{e}}_{\theta }}^{2}/N_{s}$ is the variance of the sample mean distribution.

Being that the lower the better, the value of ${\bar{e}}_{\theta }$ gives us a measure of how well an algorithm estimates the orientation. We will consider that the performance of an algorithm A is better than the performance of other algorithm B if ${\bar{e}}_{\theta }\left(A\right)<{\bar{e}}_{\theta }\left(B\right)$ and their confidence intervals do not overlap.

### 4.2. Simulation Scheme

To compute the performance metrics we will need to generate a large number of simulations. Each independent simulation will consist of three steps: initialization, convergence, and estimation.

In the initialization step we set up the initial conditions accordingly to the chosen simulation parameters. This includes generating the initial unit quaternion ${\overset{⭑}{q}}_{0}$ from a uniform distribution in $S^{3}$ setting the initial angular velocity $\overset{⭑}{\omega }{}_{0}^{\prime }$ to zero, setting the update frequency $f_{\mathrm{update}}$ generating the variances of the process noises $\sigma_{\omega }^{2}$ and $\sigma_{v}^{2}$ from a uniform distribution in the intervals $\left(0,Q_{\max}^{\omega }\right]$ and $\left(0,Q_{\max}^{v}\right]$ respectively, and initializing the estimation algorithm. The initialization of the MEKF includes setting ${\bar{q}}_{0|0}=1$ ${\bar{\omega }}_{0|0}^{\prime }=0\;\mathrm{rad}/s$ and $P_{0|0}^{{\bar{q}}_{0|0}}=10^{2}\;I$. On the other hand, the initialization of the MUKF includes setting ${\bar{q}}_{0|-1}=1$ $e_{0|0}^{{\bar{q}}_{0|-1}}=0$ ${\bar{\omega }}_{0|0}^{\prime }={\left(1,1,1\right)}^{T}\;\mathrm{rad}/s$ and $P_{0|0}^{{\bar{q}}_{0|-1}}=10^{2}\;I$. The angular velocity is not initialized to 0 in the MUKF because it has been observed that it is sometimes necessary to “break the symmetry” for the algorithm to converge; especially when we do not apply the chart update (when we perform the “reset operation”) for the RV chart. The covariance matrices that appear in both algorithms are initialized as $Q_{n}^{\omega }=I\;{\mathrm{rads}}^{2}/s^{4}$ $Q_{n}^{v}=10^{-2}\;I\;p.d.u.$ (“p.d.u.” stands for “Procedure Defined Unit”. In the present case it depends on the definition of the vector v), $R_{n}^{\omega }=R^{\omega }\;I\;{\mathrm{rads}}^{2}/s^{2}$, $R_{n}^{v}=R^{v}\;I\;p.d.u.$, where $R^{\omega }$ and $R^{v}$ are the variances of the measurement noise that will be used in the simulation. We give this information about the measurement noise to the algorithms because it can be obtained offline, while the information about the process noise cannot. Given that a priori we cannot know how the system will behave, the values of $Q_{n}^{\omega }$ and $Q_{n}^{v}$ have been chosen according to what we understand could be normal. Choosing these values we are assuming that after a second it is normal for the angular velocity to have changed by 1rad/s and also that it is normal to find external noises added to the vector $v_{t}$ of magnitude $10^{-1}\;p.d.u.$. For the mean values we set ${\bar{q}}_{n}^{\omega }=0\;\mathrm{rads}/s^{2}$ and ${\bar{q}}^{v}=0\;p.d.u.$.

In the convergence step we keep the system in the initial orientation ${\overset{⭑}{q}}_{0}$. Simulated measurements are generated using (23) and (24). For each measurement, a different $v_{t}$ is sampled from a uniform distribution in the unit sphere of $R^{3}$. The values for each component of $q_{t}^{v}$ $r_{t}^{v}$ and $r_{t}^{\omega }$ are obtained from normal distributions with zero mean and variances $\sigma_{v}^{2}$ $R^{v}$ and $R^{\omega }$ respectively. The term $R^{T}\left(q_{t}\right)$ in (23) is obtained from the true attitude ${\overset{⭑}{q}}_{t}$, which in the convergence step takes the value of ${\overset{⭑}{q}}_{t}={\overset{⭑}{q}}_{0}$. The term $\omega_{t}^{\prime }$ in (24) is the true angular velocity, which in the convergence step takes the value $\overset{⭑}{\omega }{}_{t}^{\prime }=0$. The tested algorithm updates its state estimation until the inequality $\theta_{e}\left(t\right)<\theta_{e}^{0}$ is satisfied, where $\theta_{e}\left(t\right)$ is the value of the error (72), and $\theta_{e}^{0}$ is a parameter in the simulation. The convergence step could have been replaced by an initialization of the attitude estimated by the algorithm ${\bar{q}}_{t}$ to the real value ${\overset{⭑}{q}}_{t}$ but then it would have also been necessary to fix a certain covariance matrix. Since the metric of the space generated by each chart is different, it is difficult to set a covariance matrix that provides the same information for each chart. It seemed more natural to the authors to allow the algorithm to find the true attitude by its own means, and for the covariance matrix to converge to a value in each case.

Finally, in the estimation step we generate a random but continuous orientation sequence using a Wiener process for the angular velocity:

(75) $$\begin{array}{cc} \overset{⭑}{\omega }{}_{t}^{\prime }\; & =\;\overset{⭑}{\omega }{}_{t-\delta t}^{\prime }\;+\;n_{t}\sqrt{\delta t\;}, \end{array}$$

(76) $$\begin{array}{cc} {\overset{⭑}{q}}_{t}\; & =\;{\overset{⭑}{q}}_{t-\delta t}*\left(\begin{array}{c} \;\cos\left(\frac{\;∥\overset{⭑}{\omega }{}_{t}^{\prime }∥\;\delta t\;}{\;2\;}\right)\;\;\\ \;\frac{\;\overset{⭑}{\omega }{}_{t}^{\prime }\;}{\;∥\overset{⭑}{\omega }{}_{t}^{\prime }∥\;}\;\sin\left(\frac{\;∥\overset{⭑}{\omega }{}_{t}^{\prime }∥\;\delta t\;}{\;2\;}\right)\;\; \end{array}\right), \end{array}$$

where $n_{t}$ is a random vector whose components are sampled from a normal distribution with zero mean and variance $\sigma_{\omega }^{2}$ and δt is the simulation time step that is related to the algorithm time step Δ*t* trough dtdtsimδt=Δt being dtdtsim an integer parameter that determines the simulation updates per algorithm update. Note that we multiply $n_{t}$ by $\sqrt{\delta t\;}$ and not by δt. We do it this way so that the covariance matrix after *k* steps does not depend on the simulation time step δt. In fact, after a time T=kδt the covariance matrix of the angular velocity will have grown by $\Delta P^{\omega }=k\;I\;\sigma_{\omega }^{2}\;\delta t=I\;\sigma_{\omega }^{2}\;T$ and not by ${\left(\Delta P^{\omega }\right)}^{\prime }=k\;I\;\sigma_{\omega }^{2}\;{\left(\delta t\right)}^{2}=I\;\sigma_{\omega }^{2}\;T\;\delta t$. After each dtdtsim simulation updates, a simulated measurement is generated in the same way it was done in the convergence step, and the algorithm is updated with it. The simulation will run for a time $\mathrm{Tsim}=k^{\prime }\;\Delta t$ where $k^{\prime }$ is an integer number. This way we will perform the last algorithm update at the end of the simulation. The error (72) will be evaluated after each algorithm update, and it will be added up through the simulation to obtain the averaged error (73). After each simulation, we will obtain a sample for the computation of ${\bar{e}}_{\theta }$ and $\sigma_{{\bar{e}}_{\theta }}^{2}$. We will perform $N_{s}$ of these simulations to obtain the confidence interval (74).

### 4.3. Results

**Figure 4:**
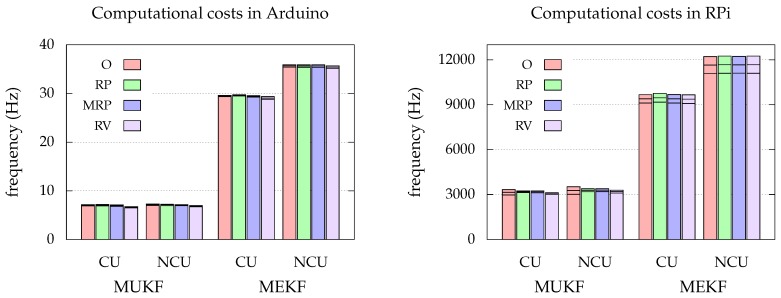
Maximum update frequency for each approach. The lines at the top represent the mean and the deviation (3σ) of the distribution of maximum update frequencies. “CU” stands for Chart Update, while “NCU” stands for No Chart Update.

In this section we present the results of the simulations. The algorithms are tested for update frequencies $f_{\mathrm{update}}=1/\Delta t$ in the interval [2,1000]Hz. This range has been chosen thinking about the possible limitations of a real system. For example, the maximum data rate of a low cost IMU is around 1000Hz. On the other hand, the update frequency may be limited by processing. The computational cost of each estimator has been evaluated in two platforms: an Arduino MEGA 2560, and a Raspberry Pi 3 Model B. The code has been written in c++. The resulting maximum update frequencies are presented in Figure 4, which indicates that the MEKF can be executed approximately 3 times faster than the MUKF.

Although the algorithms have been developed allowing a different $\Delta t_{n}$ for each update, the simulations are performed using a constant Δ*t*, and the simulation parameters depicted in Table 5.

The parameters $\theta_{e}^{0}$ Tsim, dtdtsim, and $N_{s}$ have been chosen trying to reach a compromise between the precision of the results, and the execution time of the simulation. The values for $Q_{\max}^{\omega }$ and $Q_{\max}^{v}$ have been chosen in such a way that the estimation algorithms face both normal situations ($Q_{n}^{\omega }\approx \sigma_{\omega }^{2}\;I$ and $Q_{n}^{v}\approx \sigma_{v}^{2}\;I$) and situations that were not foreseen ($Q_{n}^{\omega }\neq \sigma_{\omega }^{2}\;I$ or $Q_{n}^{v}\neq \sigma_{v}^{2}\;I$). A typical low cost IMU has $R^{\omega }\approx 10^{-4}\;{\mathrm{rad}}^{2}/s^{2}$ and $R^{v}\approx 10^{-4}\;g^{2}$. The values chosen for *R* represent an imprecise sensor ($10^{-2}$), a normal sensor ($10^{-4}$), and a precise sensor ($10^{-6}$). The value of $W_{0}$ has been chosen so that all sigma points have the same importance, but very similar results, if not identical, have been obtained for other selections of $W_{0}$.

#### 4.3.1. Chart Choice

**Figure 5:**
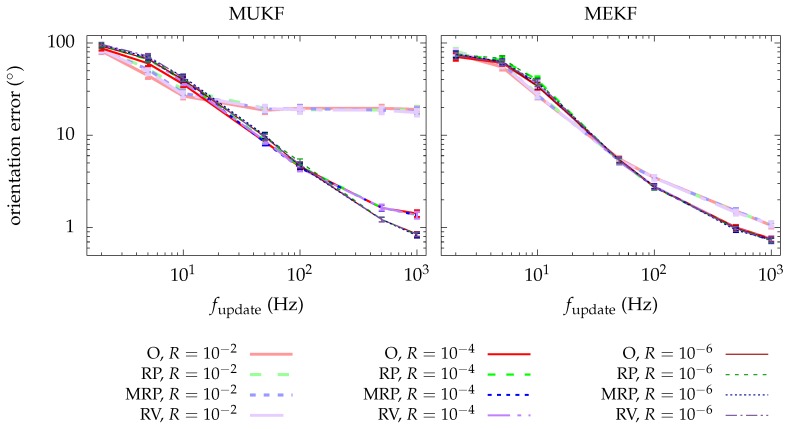
Mean of the performance metric for each approach. Results from different charts are plotted in the same graph. Results from different MKF are plotted in different graphs. Bars represent the confidence interval (3σ) for the mean computation.

The results of the simulation are presented in Figure 5. The average of the performance metric is shown along with its confidence interval for each of the selected update frequencies. The results of the MEKF and the MUKF are shown in different graphs, but drawn in the same one are the results for each chart and for a given MKF. In this way we are able to distinguish if a chart has an advantage over the others.

We observe that there is no chart that is especially advantageous. All things being equal, we would opt for the RP chart. For this chart it is not necessary to worry about the domain since it maps q and −q with the same point of $R^{3}$ and with the same T-matrix; or of the image since it is all $R^{3}$. In addition, the expressions of $\varphi^{-1}$ and the T-matrix for the MEKF are simpler for the RP chart. These computational advantages make us prefer the RP chart over the others.

#### 4.3.2. MEKF vs. MUKF

**Figure 6:**
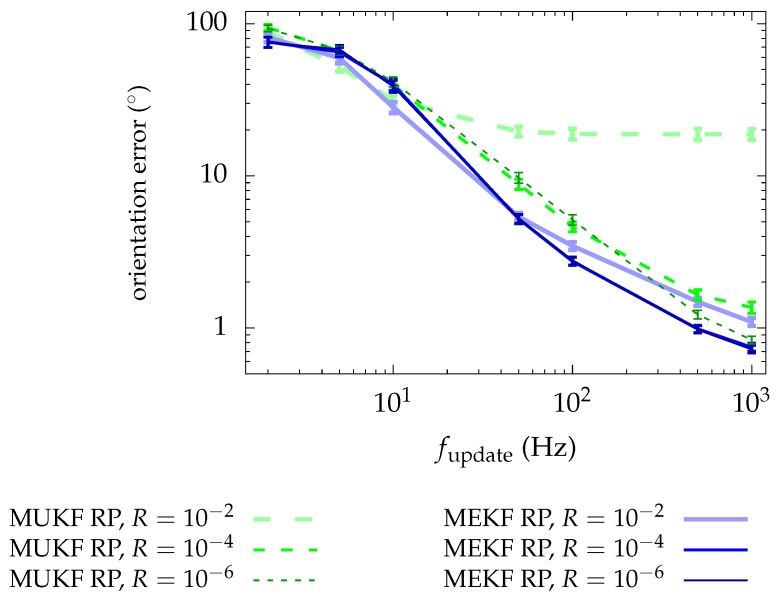
Mean of the performance metric for each MKF. Only results for the RP chart are plotted. Bars represent the confidence interval (3σ) in the mean computation.

Figure 6 also presents the results of the simulations. This time, we display on the same graph the resulting performance metrics for the MUKF and the MEKF when the RP chart is used. In this way, we can distinguish if one MKF has an advantage over the other.

We note that the MEKF performs the same or better than the MUKF. This differs from the usual experience, in which the UKF outperforms the EKF in traditional non-linear estimation applications. The fact that the charts resemble the Euclidean space near the origin (see Section 2.3) might be favoring the MEKF, since the Jacobian matrices, used to approximate the non-linear functions, are defined at that point. However, the sigma points generated for the MUKF are sampled far from the origin of the chart, where the non-linearities become notorious. We are facing a very particular scenario in which the model is approximately linear for the MEKF, while for the MUKF it is not. In addition, due to the difference in computational cost (see Figure 4), the MUKF update frequencies will generally be lower than those of the MEKF, which will imply worse accuracy in its estimations. Then, the MEKF with the RP chart seems to be our best option.

#### 4.3.3. Chart Update vs. No Chart Update

**Figure 7:**
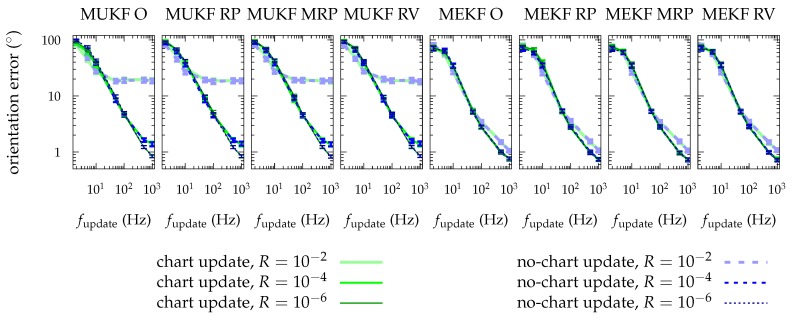
Mean of the performance metric for each approach. Results from the approach in which we apply the chart update, and those of which we do not apply it are plotted together. Bars represent the confidence interval (3σ) in the mean computation.

Figure 7 presents the results of each MKF with each chart in a different graph, but displayed in the same one are the results using the “chart update” and the results without using it.

We can observe that there is almost no difference between using the “chart update” and not using it. The concepts used in this paper have helped us to understand the mechanisms of the MKF, and ultimately to arrive to the concepts of “multiplicative update”, and of “covariance correction step” with the T-matrix definition. However, it is not necessary to apply the latest update (47b) in practice: we will obtain essentially the same accuracy in our estimations.

## 5. Conclusions

We have used concepts from manifold theory to define the expected value and the covariance matrix of a distribution in a manifold. In particular, we have defined the expected value and covariance matrix of a distribution of unit quaternions in $S^{3}$, the unit sphere in $R^{4}$, using the concept of chart. These definitions have helped us to develop Kalman filters for orientation estimation, where the attitude has been represented by a unit quaternion. They have also helped us solve the problem of the “covariance correction step”. Two estimators have been developed: one based on the EKF (the MEKF), and another based on the UKF (the MUKF). The MEKF and the MUKF have been tested in simulations, and some results have been obtained. The conclusions of the simulations are:

- There is no chart that presents a clear advantage over the others, but the RP chart has some characteristics that motivate us to prefer it.
- The MEKF is preferable to the MUKF due to its lower computational cost and its greater accuracy in orientation estimation.
- The “chart update” is not necessary for the MKF in practice.

Then, the MEKF with the RP chart and without applying the “chart update” is our best attitude estimator according to the adopted performance metric. This algorithm resembles the conventional “Multiplicative Extended Kalman Filter”, but we have obtained the MEKF without having to redefine any aspect of the classic Kalman filter. 

## Figures and Tables

**Table 1:** Main characteristics of the most used parametrizations to represent an orientation.

Representation	Parameters	Continuous	Non-Singular	Linear Evolution Equation
Euler angles	3	**✗**	**✗**	**✗**
Axis-angle	3–4	**✗**	**✗**	**✗**
Rotation matrix	9	**✓**	**✓**	**✓**
Unit quaternion	4	**✓**	**✓**	**✓**

**Table 2:** Main characteristics of the charts studied.

Chart	Domain	Image	e=φ(q)	$q=\varphi^{-1}\left(\;e\;\right)$
O	$\left\{q\in S^{3}\;:\;q_{0}\geq 0\right\}$	$\left\{e\in R^{3}\;:\;∥e∥\leq 2\right\}$	2q	$\left(\begin{array}{c} \;\sqrt{1-\frac{{∥e∥}^{2}}{4}}\;\;\\ \;e/2\;\; \end{array}\right)$
RP	$\left\{q\in S^{3}\;:\;q_{0}>0\right\}$	$R^{3}$	$2\;\frac{\;q\;}{\;q_{0}\;}$	$\frac{1}{\sqrt{4+{∥e∥}^{2}}}\left(\begin{array}{c} \;2\;\;\\ \;e\;\; \end{array}\right)$
MRP	$\left\{q\in S^{3}\;:\;q_{0}\geq 0\right\}$	$\left\{e\in R^{3}\;:\;∥e∥\leq 4\right\}$	$4\;\frac{\;q\;}{\;1+q_{0}\;}$	$\frac{1}{16+{∥e∥}^{2}}\left(\begin{array}{c} \;16-{∥e∥}^{2}\;\;\\ \;8\;e\;\; \end{array}\right)$
RV	$\left\{q\in S^{3}\;:\;q_{0}\geq 0\right\}$	$\left\{e\in R^{3}\;:\;∥e∥\leq \pi \right\}$	$2\;\hat{q}\;\arcsin\left(∥q∥\right)$	$\left(\begin{array}{c} \;\cos\left(\frac{∥e∥}{2}\right)\;\;\\ \;\hat{e}\;\sin\left(\frac{∥e∥}{2}\right)\;\; \end{array}\right)$

**Table 3:** Transition maps for the charts studied.

Chart	Transition Map $e^{\bar{p}}\left(\;e^{\bar{q}}\;\right)$
O	${\bar{\delta }}_{0}\;e^{\bar{q}}\;-\;\sqrt{4-∥e^{\bar{q}}{∥}^{2}\;}\;\bar{\delta }\;-\;\bar{\delta }\times e^{\bar{q}}$
RP	$2\;\frac{\;{\bar{\delta }}_{0}\;e^{\bar{q}}\;-\;2\;\bar{\delta }\;-\;\bar{\delta }\times e^{\bar{q}}\;}{\;2\;{\bar{\delta }}_{0}\;+\;\bar{\delta }\cdot e^{\bar{q}}\;}$
MRP	$4\;\frac{\;8\;{\bar{\delta }}_{0}\;e^{\bar{q}}\;-\;(16-∥e^{\bar{q}}{∥}^{2})\;\bar{\delta }\;-\;8\;\bar{\delta }\times e^{\bar{q}}\;}{\;16+∥e^{\bar{q}}{∥}^{2}\;+\;{\bar{\delta }}_{0}\;(16-∥e^{\bar{q}}{∥}^{2})\;+\;8\;\bar{\delta }\cdot e^{\bar{q}}\;}$
RV	$2\;\frac{\;\delta^{\bar{p}}\;}{\;∥\delta^{\bar{p}}∥\;}\;\arcsin∥\delta^{\bar{p}}∥$, with $\delta^{\bar{p}}\;=\;{\bar{\delta }}_{0}\;{\hat{e}}^{\bar{q}}\;\sin\left(\frac{∥e^{\bar{q}}∥}{2}\right)-\cos\left(\frac{∥e^{\bar{q}}∥}{2}\right)\bar{\delta }-\bar{\delta }\times {\hat{e}}^{\bar{q}}\;\sin\left(\frac{∥e^{\bar{q}}∥}{2}\right)$

**Table 4:** T-matrices for the transition maps of the charts studied.

Chart	$T(\;\bar{\delta }\;)$ Matrix	Domain
O	${\bar{\delta }}_{0}\;I\;-\;{\left[\;\bar{\delta }\;\right]}_{\times }+\frac{\;\bar{\delta }\;{\bar{\delta }}^{T}\;}{{\bar{\delta }}_{0}}$	$\left\{\;\bar{\delta }\in S^{3}\;\;:\;\;{\bar{\delta }}_{0}>0\;\right\}$
RP	${\bar{\delta }}_{0}\;\left(\;{\bar{\delta }}_{0}\;I\;-\;{\left[\;\bar{\delta }\;\right]}_{\times }\;\right)$	$\left\{\;\bar{\delta }\in S^{3}\;\;:\;\;{\bar{\delta }}_{0}\neq 0\;\right\}$
MRP	$\frac{1}{2}\;\left[\;\left(1+{\bar{\delta }}_{0}\right)\left(\;{\bar{\delta }}_{0}\;I\;-\;{\left[\;\bar{\delta }\;\right]}_{\times }\;\right)+\bar{\delta }\;{\bar{\delta }}^{T}\;\right]$	$\left\{\;\bar{\delta }\in S^{3}\;\;:\;\;{\bar{\delta }}_{0}\geq 0\;\right\}$
RV	$\left[\;{\bar{\delta }}_{0}\;\left(\;I\;-\;\hat{\bar{\delta }}\;{\hat{\bar{\delta }}}^{T}\;\right)\;-\;{\left[\;\bar{\delta }\;\right]}_{\times }\;\right]\;\frac{∥\bar{\delta }∥}{\;\arcsin∥\bar{\delta }∥\;}+\hat{\bar{\delta }}\;{\hat{\bar{\delta }}}^{T}$	$\left\{\;\bar{\delta }\in S^{3}\;\;:\;\;{\bar{\delta }}_{0}\geq 0\;,\;∥\bar{\delta }∥\neq 0\;\right\}$

**Table 5:** Parameters used in the simulations.

Parameter	Value
$\theta_{e}^{0}$	$1^{\circ }$
Tsim	10s
dtdtsim	100
$N_{s}$	1000
$Q_{\max}^{\omega }$	$10^{2}\;{\mathrm{rads}}^{2}/s^{3}$
$Q_{\max}^{v}$	1p.d.u.
*R*	$\left\{\;10^{-2}\;,\;10^{-4}\;,\;10^{-6}\;\right\}$
$R^{\omega }$	$R\;{\mathrm{rads}}^{2}/s^{2}$
$R^{v}$	Rp.d.u.
$W_{0}$	1/25

## Supplementary Materials

The following are available online at http://www.mdpi.com/1424-8220/19/1/149/s1: SupplementaryMaterials.zip.

## Abbreviations

The following abbreviations are used in this manuscript:

EKF	Extended Kalman Filter
UKF	Unscented Kalman Filter
MKF	Manifold Kalman Filter
MEKF	Manifold Extended Kalman Filter
MUKF	Manifold Unscented Kalman Filter
O	Orthographic
RP	Rodrigues Parameters
MRP	Modified Rodrigues Parameters
RV	Rotation Vector

## Appendix A. Derivation of Transition Maps

This appendix contains the derivation of the transition map for each chart.

### Appendix A.1. Orthographic

Using the inverse of the transformation that defines the chart, $\varphi^{-1}$,

(A1) $$\delta^{\bar{q}}=\left(\begin{array}{c} \;\sqrt{1\;-\;\frac{∥e^{\bar{q}}{∥}^{2}}{4}\;}\;\;\\ \;e^{\bar{q}}/2\;\; \end{array}\right).$$

Introducing (A1) into (19),

(A2) $$\delta^{\bar{p}}={\bar{\delta }}^{*}*\delta^{\bar{q}}=\left(\begin{array}{c} \;{\bar{\delta }}_{0}\;\sqrt{1\;-\;\frac{∥e^{\bar{q}}{∥}^{2}}{4}\;}+\bar{\delta }\cdot \frac{e^{\bar{q}}}{2}\;\;\\ \;{\bar{\delta }}_{0}\;\frac{e^{\bar{q}}}{2}\;-\;\sqrt{1\;-\;\frac{∥e^{\bar{q}}{∥}^{2}}{4}\;}\;\bar{\delta }\;-\;\bar{\delta }\times \frac{e^{\bar{q}}}{2}\;\; \end{array}\right).$$

Finally, applying the chart definition,

(A3) $$e^{\bar{p}}=2\;\delta^{\bar{p}}={\bar{\delta }}_{0}\;e^{\bar{q}}\;-\;\sqrt{4\;-\;∥e^{\bar{q}}{∥}^{2}\;}\;\bar{\delta }\;-\;\bar{\delta }\times e^{\bar{q}}.$$

### Appendix A.2. Rodrigues Parameters

Using the inverse of the transformation that defines the chart, $\varphi^{-1}$,

(A4) $$\delta^{\bar{q}}=\frac{1}{\;\sqrt{4\;+\;∥e^{\bar{q}}{∥}^{2}\;}\;}\left(\begin{array}{c} \;2\;\;\\ \;e^{\bar{q}}\;\; \end{array}\right).$$

Introducing (A4) into (19),

(A5) $$\delta^{\bar{p}}={\bar{\delta }}^{*}*\delta^{\bar{q}}=\frac{1}{\;\sqrt{4\;+\;∥e^{\bar{q}}{∥}^{2}\;}\;}\left(\begin{array}{c} \;2\;{\bar{\delta }}_{0}+\bar{\delta }\cdot e^{\bar{q}}\;\;\\ \;{\bar{\delta }}_{0}\;e^{\bar{q}}\;-\;2\;\bar{\delta }\;-\;\bar{\delta }\times e^{\bar{q}}\;\; \end{array}\right).$$

Finally, applying the chart definition,

(A6) $$e^{\bar{p}}=2\;\frac{\;\delta^{\bar{p}}\;}{\;\delta_{0}^{\bar{p}}\;}=2\;\frac{\;{\bar{\delta }}_{0}\;e^{\bar{q}}-2\;\bar{\delta }-\bar{\delta }\times e^{\bar{q}}\;}{\;2\;{\bar{\delta }}_{0}+\bar{\delta }\cdot e^{\bar{q}}\;}.$$

### Appendix A.3. Modified Rodrigues Parameters

Using the inverse of the transformation that defines the chart, $\varphi^{-1}$,

(A7) $$\delta^{\bar{q}}=\frac{1}{\;16+∥e^{\bar{q}}{∥}^{2}\;}\left(\begin{array}{c} \;16\;-\;∥e^{\bar{q}}{∥}^{2}\;\;\\ \;8\;e^{\bar{q}}\;\; \end{array}\right).$$

Introducing (A7) into (19),

(A8) $$\delta^{\bar{p}}={\bar{\delta }}^{*}*\delta^{\bar{q}}=\frac{1}{\;16+∥e^{\bar{q}}{∥}^{2}\;}\left(\begin{array}{c} \;{\bar{\delta }}_{0}\;\left(16-∥e^{\bar{q}}{∥}^{2}\right)+8\;\bar{\delta }\cdot e^{\bar{q}}\;\;\\ \;8\;{\bar{\delta }}_{0}\;e^{\bar{q}}\;-\;\left(16-∥e^{\bar{q}}{∥}^{2}\right)\;\bar{\delta }\;-\;8\;\bar{\delta }\times e^{\bar{q}}\;\; \end{array}\right)\;.$$

Finally, applying the chart definition,

(A9) $$e^{\bar{p}}=4\;\frac{\delta^{\bar{p}}}{\;1\;+\;\delta_{0}^{\bar{p}}\;}=4\;\frac{\;8\;{\bar{\delta }}_{0}\;e^{\bar{q}}-\left(16-∥e^{\bar{q}}{∥}^{2}\right)\;\bar{\delta }-8\;\bar{\delta }\times e^{\bar{q}}\;}{\;16+∥e^{\bar{q}}{∥}^{2}+{\bar{\delta }}_{0}\;\left(16-∥e^{\bar{q}}{∥}^{2}\right)+8\;\bar{\delta }\cdot e^{\bar{q}}\;}.$$

### Appendix A.4. Rotation Vector

Using the inverse of the transformation that defines the chart, $\varphi^{-1}$,

(A10) $$\delta^{\bar{q}}=\left(\begin{array}{c} \;\cos\left(\frac{∥e^{\bar{q}}∥}{2}\right)\;\;\\ \;{\hat{e}}^{\bar{q}}\;\sin\left(\frac{∥e^{\bar{q}}∥}{2}\right)\;\; \end{array}\right).$$

Introducing (A10) into (19),

(A11) $$\delta^{\bar{p}}={\bar{\delta }}^{*}*\delta^{\bar{q}}=\left(\begin{array}{c} \;{\bar{\delta }}_{0}\;\cos\left(\frac{∥e^{\bar{q}}∥}{2}\right)+\bar{\delta }\cdot {\hat{e}}^{\bar{q}}\;\sin\left(\frac{∥e^{\bar{q}}∥}{2}\right)\;\;\\ \;{\bar{\delta }}_{0}\;{\hat{e}}^{\bar{q}}\;\sin\left(\frac{∥e^{\bar{q}}∥}{2}\right)\;-\;\cos\left(\frac{∥e^{\bar{q}}∥}{2}\right)\;\bar{\delta }\;-\;\bar{\delta }\times {\hat{e}}^{\bar{q}}\;\sin\left(\frac{∥e^{\bar{q}}∥}{2}\right)\;\; \end{array}\right)\;.$$

Finally, applying the chart definition,

(A12) $$e^{\bar{p}}=2\;\frac{\;\delta^{\bar{p}}\;}{\;∥\delta^{\bar{p}}∥\;}\;\arcsin∥\delta^{\bar{p}}∥,$$

with

(A13) $$\begin{array}{cc} \delta^{\bar{p}}\; & =\;{\bar{\delta }}_{0}\;{\hat{e}}^{\bar{q}}\;\sin\left(\frac{∥e^{\bar{q}}∥}{2}\right)\;-\;\cos\left(\frac{∥e^{\bar{q}}∥}{2}\right)\;\bar{\delta }\;-\;\bar{\delta }\times {\hat{e}}^{\bar{q}}\;\sin\left(\frac{∥e^{\bar{q}}∥}{2}\right). \end{array}$$

Note that all transition maps are expressed using the $\bar{\delta }$ quaternion. Given that $\bar{e}=\varphi \left(\bar{\delta }\right)$ we could also have expressed them using $\bar{e}$ which is what we get after applying the Kalman update (41). However, our choice makes transition maps to take a simpler form. In addition, having to compute the quaternion $\bar{\delta }$ to perform (43c), this choice does not imply a computational overhead.

## Appendix B. Details in the Derivation of the MEKF

This appendix contains the details in the derivation of the Manifold Extended Kalman Filter used in this study.

### Appendix B.1. State Prediction

This subsection contains the derivation of the equations for the state prediction.

#### Appendix B.1.1. Evolution of the Expected Value of the State

Taking expected values in Equation (21) we obtain

(A14) $$\frac{\;d\;{\bar{\omega }}^{\prime }\;}{\;dt\;}={\bar{q}}^{\omega }\;\Rightarrow \;{\bar{\omega }}^{\prime }\left(t\right)={\bar{\omega }}_{0}^{\prime }\;+\;{\bar{q}}_{t}^{\omega }\;t,$$

with ${\bar{q}}_{t}^{\omega }$ the expected value of the random variable $q^{\omega }$ at time *t*. Doing ${\bar{\omega }}_{0}^{\prime }={\bar{\omega }}_{n-1|n-1}^{\prime }$ we arrive at

(A15) $${\bar{\omega }}_{t|n-1}^{\prime }={\bar{\omega }}_{n-1|n-1}^{\prime }\;+\;{\bar{q}}_{t}^{\omega }\;t.$$

On the other hand, approximating (22) with its Taylor series up to first order around the current state $\left(\;\bar{q}\;,\;{\bar{\omega }}^{\prime }\;\right)$, and taking its expected value we obtain

(A16) $$E\left[\frac{\;d\;q(t)\;}{\;dt\;}\right]\;\approx \;\frac{1}{2}\;\bar{q}\left(t\right)*{\bar{\omega }}^{\prime }\left(t\right).$$

This differential equation has no general closed solution. But if we assume that the expected value of the process noise ${\bar{q}}^{\omega }\left(t\right)$ is zero when $t\in (t_{n-1},t_{n})$, so that ${\bar{\omega }}^{\prime }\left(t\right)$ is constant in that interval, then we will have the matrix differential equation

(A17) $$\frac{\;d\;\bar{q}\left(t\right)\;}{\;dt\;}={\overset{ˇ}{\Omega }}_{n}\;\bar{q}\left(t\right),$$

with

(A18) $${\overset{ˇ}{\Omega }}_{n}:=\frac{1}{2}{\left(\begin{array}{ccccccc} 0 & \; & -{\bar{\omega }}_{1}^{\prime } & \; & -{\bar{\omega }}_{2}^{\prime } & \; & -{\bar{\omega }}_{3}^{\prime }\\ {\bar{\omega }}_{1}^{\prime } & \; & 0 & \; & {\bar{\omega }}_{3}^{\prime } & \; & -{\bar{\omega }}_{2}^{\prime }\\ {\bar{\omega }}_{2}^{\prime } & \; & -{\bar{\omega }}_{3}^{\prime } & \; & 0 & \; & {\bar{\omega }}_{1}^{\prime }\\ {\bar{\omega }}_{3}^{\prime } & \; & {\bar{\omega }}_{2}^{\prime } & \; & -{\bar{\omega }}_{1}^{\prime } & \; & 0 \end{array}\right)}_{\;n|n-1}.$$

This differential equation has the solution

(A19) $$\bar{q}\left(t\right)=e^{\overset{ˇ}{\Omega }\;t}\;{\bar{q}}_{0},$$

where ${\bar{q}}_{0}$ represents the initial conditions. After taking ${\bar{q}}_{0}={\bar{q}}_{n-1|n-1}$, we obtain the prediction ${\bar{q}}_{t|n-1}$, that can be expressed using the quaternion product as

(A20) $${\bar{q}}_{t|n-1}={\bar{q}}_{n-1|n-1}*\delta_{t}^{\omega }={\bar{q}}_{n-1|n-1}*\left(\begin{array}{c} \;\cos\left(\frac{\;∥{\bar{\omega }}_{t|n-1}^{\prime }∥\Delta t}{2}\right)\;\;\\ \;\frac{\;{\bar{\omega }}_{t|n-1}^{\prime }\;}{\;∥{\bar{\omega }}_{t|n-1}^{\prime }∥\;}\;\sin\left(\frac{\;∥{\bar{\omega }}_{t|n-1}^{\prime }∥\Delta t}{2}\right)\;\; \end{array}\right),$$

with $\Delta t=t-t_{n-1}$.

#### Appendix B.1.2. Evolution of the State Covariance Matrix

For a continuous nonlinear system of the form

(A21) $$\frac{\;d\;x\;}{\;dt\;}=f\left(\;x\;,\;t\;\right)\;+\;g\left(\;q\;\right),$$

we know [18] that the covariance matrix satisfies the following differential equation:

(A22) $$\frac{\;d\;P\;}{\;dt\;}=F\;P\;+\;P\;F^{T}\;+\;G\;Q\;G^{T},$$

where $F=\frac{\;\partial \;f\;}{\;\partial x\;}$, and $G=\frac{\;\partial \;g\;}{\;\partial q\;}$. This is so because the evolution equation for $\Delta x=x-\bar{x}$ is approximately given by:

(A23) $$\frac{\;d\;x\;}{\;dt\;}\;\approx \;\frac{\;d\;\bar{x}\;}{\;dt\;}+{\left.\frac{\;\partial \;f\;}{\;\partial x\;}\right|}_{x=\bar{x}}\left(\;x\;-\;\bar{x}\;\right)+{\left.\frac{\;\partial \;g\;}{\;\partial q\;}\right|}_{q=\bar{q}}\left(\;q\;-\;\bar{q}\;\right),$$

and P is defined as $P=E\left[\Delta x\;{\left(\Delta x\right)}^{T}\;\right]$. However, we have a different definition for P:

(A24) $$P=E\left[\;\left(\begin{array}{c} \;e^{\bar{q}}-{\bar{e}}^{\bar{q}}\;\;\\ \;\omega^{\prime }-{\bar{\omega }}^{\prime }\;\; \end{array}\right)\;{\left(\begin{array}{c} \;e^{\bar{q}}-{\bar{e}}^{\bar{q}}\;\;\\ \;\omega^{\prime }-{\bar{\omega }}^{\prime }\;\; \end{array}\right)}^{T}\;\right].$$

Then we need to find the evolution equation for $e^{\bar{q}}$. Recall that we are assuming ${\bar{e}}^{\bar{q}}=0$ at the beginning of the iteration. Knowing that any quaternion in the unit sphere can be expressed as a deviation from a central quaternion $\bar{q}$ as $q=\bar{q}*\delta$, and using the differential Equations (22) and (A16), we can find a differential equation for the quaternion δ:

(A25a) $$\begin{array}{cc} q\; & =\;\bar{q}*\delta \;\Rightarrow \end{array}$$

(A25b) $$\begin{array}{cc} \Rightarrow \;\dot{q}\; & =\;\dot{\bar{q}}*\delta \;\;+\;\;\bar{q}*\dot{\delta }\;\Rightarrow \end{array}$$

(A25c) $$\begin{array}{cc} \Rightarrow \;\frac{1}{2}\;q*\omega^{\prime }\; & \approx \;\frac{1}{2}\;\bar{q}*{\bar{\omega }}^{\prime }*\delta \;\;+\;\;\bar{q}*\dot{\delta }, \end{array}$$

where a dot over a symbol represents time derivative, and we have obviated the time dependence. Isolating the time derivative $\dot{\delta }$,

(A26a) $$\begin{array}{cc} \dot{\delta }\; & \approx \;\frac{1}{2}\;\overset{\delta }{\overset{︷}{{\bar{q}}^{*}*q}}*\omega^{\prime }\;-\;\frac{1}{2}\;\overset{1}{\overset{︷}{{\bar{q}}^{*}*\bar{q}}}*{\bar{\omega }}^{\prime }*\delta \;= \end{array}$$

(A26b) $$\begin{array}{c} =\;\frac{1}{2}\;\left[\;\delta *\omega^{\prime }\;-\;{\bar{\omega }}^{\prime }*\delta \;\right]\;= \end{array}$$

(A26c) $$\begin{array}{c} =\;\frac{1}{2}\;\left[\;\left(\begin{array}{c} \delta_{0}\\ \delta \end{array}\right)*\left(\begin{array}{c} 0\\ \omega^{\prime } \end{array}\right)\;-\;\left(\begin{array}{c} 0\\ {\bar{\omega }}^{\prime } \end{array}\right)*\left(\begin{array}{c} \delta_{0}\\ \delta \end{array}\right)\;\right]\;= \end{array}$$

(A26d) $$\begin{array}{c} =\;\frac{1}{2}\;\left(\begin{array}{c} -\left(\;\omega^{\prime }\;-\;{\bar{\omega }}^{\prime }\;\right)\cdot \delta \;\;\\ \;\delta_{0}\;\left(\;\omega^{\prime }\;-\;{\bar{\omega }}^{\prime }\;\right)\;-\;\left(\;\omega^{\prime }\;+\;{\bar{\omega }}^{\prime }\;\right)\times \delta \;\; \end{array}\right)\;= \end{array}$$

(A26e) $$\begin{array}{c} =\;\frac{1}{2}\;\left(\begin{array}{c} -\Delta \omega^{\prime }\cdot \delta \;\;\\ \;\delta_{0}\Delta \omega^{\prime }\;-\;\left(\;2\;{\bar{\omega }}^{\prime }+\Delta \omega^{\prime }\;\right)\times \delta \end{array}\right). \end{array}$$

Knowing that, for each of our charts, the δ quaternion can be approximated by (10) as e→0, then we can obtain an approximate differential equation for a point e expressed in the $\bar{q}$-centered chart. Note that we have not explicitly denoted $e^{\bar{q}}$ or $\delta^{\bar{q}}$. This will be assumed implicitly, since these quantities will always be expressed in the $\bar{q}$-centered chart in this appendix. Using the chain rule for a time derivative and expression (A26e),

(A27a) e˙i=∑j∂ei∂δj︷2δij≈2δij∂δj∂t︷δ˙j≡

(A27b) $$\begin{array}{cc} \equiv \;\dot{e}\; & \approx \;\delta_{0}\;\Delta \omega^{\prime }\;-\;\left(\;2\;{\bar{\omega }}^{\prime }+\Delta \omega^{\prime }\;\right)\times \delta \;\approx \end{array}$$

(A27c) $$\begin{array}{c} \approx \;\left(1-\frac{{\;∥e∥}^{2}\;}{\;8\;}\right)\Delta \omega^{\prime }\;-\;\left(\;2\;{\bar{\omega }}^{\prime }+\Delta \omega^{\prime }\;\right)\times \frac{\;e\;}{\;2\;}. \end{array}$$

Then, the first order approximation to differential Equation (A27c) would be

(A28) $$\dot{e}\;\approx \;\Delta \omega^{\prime }-{\bar{\omega }}^{\prime }\times e.$$

On the other hand, combining Equations (21) and (A14) we obtain

(A29) $$\frac{\;d\;\Delta \omega^{\prime }\;}{\;dt\;}=\frac{\;d\;(\omega^{\prime }-{\bar{\omega }}^{\prime })\;}{\;dt\;}=q^{\omega }-{\bar{q}}^{\omega }=q^{\omega }.$$

Summarizing,

(A30) $$\frac{\;d\;}{\;dt\;}\left(\begin{array}{c} \;e\;\;\\ \Delta \omega^{\prime } \end{array}\right)\;\approx \;\left(\begin{array}{cc} \;\;-{\left[{\bar{\omega }}^{\prime }\right]}_{\times }\; & \;I\;\;\\ \;\;0\; & \;0\;\; \end{array}\right)\;\left(\begin{array}{c} \;e\;\;\\ \Delta \omega^{\prime }\;\; \end{array}\right)+\left(\begin{array}{c} \;0\;\;\\ \Delta q^{\omega }\;\; \end{array}\right),$$

therefore matrices F, G, and Q in (A22) are in our case

(A31) $$\begin{array}{cc} F\; & =\;\left(\begin{array}{cc} \;\;-{\left[{\bar{\omega }}^{\prime }\right]}_{\times }\; & \;I\;\;\\ \;\;0\; & \;0\;\; \end{array}\right), \end{array}$$

(A32) $$\begin{array}{cc} G\; & =\;I, \end{array}$$

(A33) $$\begin{array}{cc} Q\; & =\;\left(\begin{array}{cc} \;\;0\; & \;0\;\;\\ \;\;0\; & \;E[\Delta q^{\omega }\;{\left(\Delta q^{\omega }\right)}^{T}\;]\;\; \end{array}\right). \end{array}$$

We are now in a position to solve the differential Equation (A22). Let us consider its homogeneous version first:

(A34) $$\frac{\;d\;P_{H}\;}{\;dt\;}=F\;P_{H}\;+\;P_{H}\;F^{T},$$

which has as solution

(A35) $$P_{H}=e^{F\;t}\;C_{0}\;e^{F^{T}\;t}.$$

Taking into account the definition of matrix exponential, and after computing the powers of F we obtain

(A36) $$e^{F\;t}=\left(\begin{array}{cc} \;\;\sum_{n=0}^{\infty }\frac{\;{\left(-\Omega \right)}^{n}\;t^{n}\;}{n!}\; & \;\sum_{n=1}^{\infty }\frac{\;{\left(-\Omega \right)}^{n-1}\;t^{n}\;}{n!}\;\;\\ \;\;0\; & \;I\;\; \end{array}\right)\;\approx \;\left(\begin{array}{cc} \;\;R^{T}\left(\;\delta^{\omega }\;\right)\; & \;I\;t\;\;\\ \;\;0\; & \;I\;\; \end{array}\right),$$

where we have denoted $\Omega ={\left[{\bar{\omega }}^{\prime }\right]}_{\times }$, and $\delta^{\omega }=\left(\;\cos\frac{∥{\bar{\omega }}^{\prime }∥\;t}{2}\;,\;\frac{{\bar{\omega }}^{\prime }}{∥{\bar{\omega }}^{\prime }∥}\;\sin\frac{∥{\bar{\omega }}^{\prime }∥\;t}{2}\;\right)$. We also have assumed that *t* takes small values so we can approximate the infinite sums truncating in the first term. To find the solution of the non-homogeneous differential equation we use the *variation of constants method*:

(A37) $$\begin{array}{cc} P\; & =\;e^{F\;t}\;C\left(t\right)\;e^{F^{T}\;t}\;\Rightarrow \end{array}$$

(A38) $$\begin{array}{cc} \Rightarrow \;\frac{\;d\;P\;}{\;dt\;}\; & =\;F\;e^{F\;t}\;C\left(t\right)\;e^{F^{T}\;t}\;\;+\;\;e^{F\;t}\;C\left(t\right)\;e^{F^{T}\;t}\;F^{T}\;\;+\;\;e^{F\;t}\;\frac{\;d\;C(t)\;}{\;dt\;}\;e^{F^{T}\;t}\;= \end{array}$$

(A39) $$\begin{array}{c} =\;F\;P\;\;+\;\;P\;F^{T}\;\;+\;\;e^{F\;t}\;\frac{\;d\;C(t)\;}{\;dt\;}\;e^{F^{T}\;t}. \end{array}$$

Identifying terms with (A22) we obtain that

(A40) $$\begin{array}{cc} e^{F\;t}\;\frac{\;d\;C(t)\;}{\;dt\;}\;e^{F^{T}\;t}\; & =\;G\;Q\;G^{T}\;\Rightarrow \end{array}$$

(A41) $$\begin{array}{cc} \Rightarrow \;\frac{\;d\;C(t)\;}{\;dt\;}\; & =\;e^{-F\;t}\;Q\;e^{-F^{T}\;t}\;=\;\sum_{n=0}^{\infty }\sum_{m=0}^{\infty }\frac{\;{\left(-F\right)}^{n}\;t^{n}\;}{n!}\;Q\;\frac{\;{\left(-F^{T}\right)}^{m}\;t^{m}\;}{m!}\;\Rightarrow \end{array}$$

(A42) $$\begin{array}{cc} \Rightarrow \;C(t)\; & =\;C_{0}\;\;+\;\;\sum_{n=0}^{\infty }\sum_{m=0}^{\infty }\frac{\;{\left(-F\right)}^{n}\;}{n!}\;Q\;\frac{\;{\left(-F^{T}\right)}^{m}\;}{m!}\;\frac{\;t^{n+m+1}\;}{\;n+m+1\;}. \end{array}$$

Finally, truncating the summation in (A42) at the first non-zero elements, and inserting the result into (A37), we obtain (32) where we have identified $C_{0}=P\left(0\right)$ through the initial conditions.

### Appendix B.2. Measurement Prediction

This subsection contains the derivation of the equations for the measurement prediction.

#### Appendix B.2.1. Expected Value of the Measurement Prediction

Taking expected values on (24), and assuming ${\bar{r}}_{t}^{\omega }=0$ we arrive at (35). On the other hand, approximating (23) with its Taylor series up to first order around the current estimation of the state $\left(\;\bar{q}\;,\;{\bar{\omega }}^{\prime }\;\right)$, taking its expected value, and assuming ${\bar{r}}_{t}^{v}=0$ we obtain (34).

#### Appendix B.2.2. Covariance Matrix of the Measurement Prediction

In order to find the covariance matrix of the measurement prediction we need the linear approximation of the vector measurement around the point $x_{0}:=\left(\;e=0\;,\;q^{v}={\bar{q}}^{v}\;,\;r^{v}={\bar{r}}^{v}\;\right)$:

(A43) $$\begin{array}{cc} v^{m}\;\approx \;{\bar{v}}^{m}\;\; & +\;\;{\left.\frac{\;\partial \;v^{m}\;}{\;\partial e\;}\right|}_{x_{0}}\;e\;\;+\;\;{\left.\frac{\;\partial \;v^{m}\;}{\;\partial q^{v}\;}\right|}_{x_{0}}\;\left(q^{v}-{\bar{q}}^{v}\right)\;\;+\;\;{\left.\frac{\;\partial \;v^{m}\;}{\;\partial r^{v}\;}\right|}_{x_{0}}\;\left(r^{v}-{\bar{r}}^{v}\right). \end{array}$$

It is direct to identify

(A44) $$\begin{array}{cc} {\left.\frac{\;\partial \;v^{m}\;}{\;\partial q^{v}\;}\right|}_{x_{0}}\; & =\;R^{T}\left(\;\bar{q}\;\right), \end{array}$$

(A45) $$\begin{array}{cc} {\left.\frac{\;\partial \;v^{m}\;}{\;\partial r^{v}\;}\right|}_{x_{0}}\; & =\;I. \end{array}$$

On the other hand, rewriting (23) as

(A46) $$\begin{array}{cc} v^{m}\; & =\;\delta^{*}*{\bar{q}}^{*}*\left(\;q^{v}+v\;\right)*\bar{q}*\delta \;+\;r^{v}\;\equiv \end{array}$$

(A47) $$\begin{array}{cc} \equiv \;v^{m}\; & =\;R^{T}\left(\delta \right)\;R^{T}\left(\bar{q}\right)\;\left(\;q^{v}+v\;\right)\;+\;r^{v}, \end{array}$$

and noting that setting e=0 is equivalent to do δ=1,

(A48) $${\left.\frac{\;\partial \;v_{i}^{m}\;}{\;\partial e_{j}\;}\right|}_{x_{0}}\;=\;\sum_{k}{\left.\frac{\;\partial \;v_{i}^{m}\;}{\;\partial \delta_{k}\;}\right|}_{x_{0}}{\left.\frac{\;\partial \;\delta_{k}\;}{\;\partial e_{j}\;}\right|}_{e=0}\;=\;\sum_{kl}{\left.\frac{\;\partial \;R_{il}^{T}\left(\delta \right)\;}{\;\partial \delta_{k}\;}\right|}_{\delta =1}{\left[\;R^{T}\left(\bar{q}\right)\;\left(\;{\bar{q}}^{v}\;+\;v\;\right)\;\right]}_{l}{\left.\frac{\;\partial \;\delta_{k}\;}{\;\partial e_{j}\;}\right|}_{e=0}\;.$$

Now, recalling (5) we have

(A49) $${\left.\frac{\;\partial \;R^{T}\left(\delta \right)\;}{\;\partial \delta_{k}\;}\right|}_{\delta =1}=2\;\left(\begin{array}{ccc} \;\;0 & \delta_{3k} & -\delta_{2k}\;\;\\ \;\;-\delta_{3k} & 0 & \delta_{1k}\;\;\\ \;\;\delta_{2k} & -\delta_{1k} & 0\;\; \end{array}\right)\;\equiv \;-\;2\sum_{n}\varepsilon_{inl}\;\delta_{nk},$$

with $\varepsilon_{inl}$ the Levi-Civita symbol, and $\delta_{nk}$ the Kronecker delta. Recalling (10) we also have

(A50) $$\frac{\;\partial \;\delta \;}{\;\partial e_{j}\;}={\left.\left(\begin{array}{c} \;-e_{j}/4\;\;\\ \;\delta_{1j}/2\;\;\\ \;\delta_{2j}/2\;\;\\ \;\delta_{3j}/2\;\; \end{array}\right)\right|}_{e=0}\;\equiv \;\left(1-\delta_{0k}\right)\;\delta_{kj}/2.$$

Then, introducing in (A48) Equations (A49), (34), and (A50),

(A51) $${\left.\frac{\;\partial \;v_{i}^{m}\;}{\;\partial e_{j}\;}\right|}_{x_{0}}\;\approx \;-\sum_{kln}\varepsilon_{inl}\;\delta_{nk}\;{\bar{v}}_{l}^{m}\;\left(1-\delta_{0k}\right)\;\delta_{kj}=\sum_{l}\varepsilon_{ilj}\;{\bar{v}}_{l}^{m}\;\equiv \;{\left[\;{\bar{v}}^{m}\;\right]}_{\;\times },$$

where we have used $\varepsilon_{ijl}=-\varepsilon_{ilj}$. Finally, assuming the independence of the random variables $x_{t}={\left(\;e_{t}\;,\;\omega_{t}^{\prime }\;\right)}^{T}$, $q_{t}^{v}$, $r_{t}^{v}$, and $r_{t}^{\omega }$, and computing the covariance matrix $S_{t}=E\left[\;\left(z_{t}-{\bar{z}}_{t}\right){\left(z_{t}-{\bar{z}}_{t}\right)}^{T}\;\right]$ with $z_{t}={\left(\;v_{t}^{m}\;,\;\omega_{t}^{m}\;\right)}^{T}$ and (A43), we arrive at (37) and (38).

## Appendix C. Derivation of the T-matrices

This appendix contains the derivation of the T-matrix for each chart.

### Appendix C.1. Orthographic

Our transition map (A3) can be written as

(A52) $$\begin{array}{cc} e_{i}^{\bar{p}}\left(e^{\bar{q}}\right)\; & =\;{\bar{\delta }}_{0}\;e_{i}^{\bar{q}}\;-\;\sqrt{4\;-\;\sum_{k}{\left(e_{k}^{\bar{q}}\right)}^{2}\;}\;{\bar{\delta }}_{i}\;-\;\sum_{lm}\varepsilon_{ilm}\;{\bar{\delta }}_{l}\;e_{m}^{\bar{q}}, \end{array}$$

being $\varepsilon_{ilm}$ the Levi-Civita symbol. Finding (45) for (A52) we obtain

(A53a) $$\begin{array}{cc} {\left(T\right)}_{ij}\; & =\;{\left[\;{\bar{\delta }}_{0}\;\delta_{ij}\;-\;\frac{\;-\sum_{k}e_{k}^{\bar{q}}\;\delta_{kj}\;}{\;\sqrt{4\;-\;\sum_{k}{\left(e_{k}^{\bar{q}}\right)}^{2}\;}\;}\;{\bar{\delta }}_{i}\;-\;\sum_{lm}\varepsilon_{ilm}\;{\bar{\delta }}_{l}\;\delta_{mj}\;\right]}_{e^{\bar{q}}={\bar{e}}^{\bar{q}}}\;= \end{array}$$

(A53b) $$\begin{array}{c} =\;{\bar{\delta }}_{0}\;\delta_{ij}\;+\;\frac{{\bar{e}}_{j}^{\bar{q}}}{\;\sqrt{4\;-\;\sum_{k}{\left({\bar{e}}_{k}^{\bar{q}}\right)}^{2}\;}\;}\;{\bar{\delta }}_{i}\;-\;\sum_{l}\varepsilon_{ilj}\;{\bar{\delta }}_{l}, \end{array}$$

This expression can be rewritten in matrix form as

(A54) $$T\;=\;{\bar{\delta }}_{0}\;I\;+\;\frac{\;\bar{\delta }\;{\left({\bar{e}}^{\bar{q}}\right)}^{T}\;}{\;\sqrt{4\;-\;∥{\bar{e}}^{\bar{q}}{∥}^{2}\;}\;}\;-\;{\left[\;\bar{\delta }\;\right]}_{\times }.$$

Finally, recalling that for this chart ${\bar{\delta }}_{0}=\sqrt{1\;-\;∥{\bar{e}}^{\bar{q}}{∥}^{2}/4\;}$ and $\bar{\delta }={\bar{e}}^{\bar{q}}/2$, we arrive at the final expression

(A55) $$T\;=\;{\bar{\delta }}_{0}\;I\;+\;\frac{\;\bar{\delta }\;{\bar{\delta }}^{T}\;}{\;{\bar{\delta }}_{0}\;}\;-\;{\left[\;\bar{\delta }\;\right]}_{\times }.$$

### Appendix C.2. Rodrigues Parameters

First, let us denote the numerator of (A6) as $N(e^{\bar{q}})$, and its denominator as $D(e^{\bar{q}})$:

(A56) $$\begin{array}{cc} N\left(\;e^{\bar{q}}\;\right)\; & :=\;{\bar{\delta }}_{0}\;e^{\bar{q}}\;-\;2\;\bar{\delta }\;-\;\bar{\delta }\times e^{\bar{q}}, \end{array}$$

(A57) $$\begin{array}{cc} D\left(\;e^{\bar{q}}\;\right)\; & :=\;2\;{\bar{\delta }}_{0}+\bar{\delta }\cdot e^{\bar{q}}. \end{array}$$

Now let us evaluate (A56) at ${\bar{e}}^{\bar{q}}$:

(A58) $$N\left(\;{\bar{e}}^{\bar{q}}\;\right)\;=\;\overset{0}{\overset{︷}{{\bar{\delta }}_{0}\;\;\underset{2\;\bar{\delta }/{\bar{\delta }}_{0}}{\underset{︸}{{\bar{e}}^{\bar{q}}}}\;\;-\;2\;\bar{\delta }}}\;-\;\overset{=(\;\bar{\delta }\;∥\;{\bar{e}}^{\bar{q}}\;)}{\overset{︷}{\bar{\delta }\times {\bar{e}}^{\bar{q}}}}\;=\;0.$$

Then, the approximation of $N\left(\;e^{\bar{q}}\;\right)$ does not have terms of order O(1). This means that we will only need to approximate $D\left(e^{\bar{q}}\right)$ to the zeroth order. Any further approximation would produce, after multiplying by the linear approximation of $N\left(\;e^{\bar{q}}\;\right)$, a higher order term. Let us then calculate each approximation.

We can rewrite (A56) as

(A59) $$N_{i}\left(e^{\bar{q}}\right)\;=\;{\bar{\delta }}_{0}\;e_{i}^{\bar{q}}\;-\;2\;{\bar{\delta }}_{i}\;-\;\sum_{kl}\varepsilon_{ikl}\;{\bar{\delta }}_{k}\;e_{l}^{\bar{q}},$$

with $\varepsilon_{ikl}$ the Levi-Civita symbol. Applying (44) to (A59),

(A60a) $$\begin{array}{cc} N_{i}\left(\;e^{\bar{q}}\;\right)\; & \approx \;\sum_{j}{\left[\;{\bar{\delta }}_{0}\;\delta_{ij}\;-\;\sum_{kl}\varepsilon_{ikl}\;{\bar{\delta }}_{k}\;\delta_{lj}\;\right]}_{e^{\bar{q}}={\bar{e}}^{\bar{q}}}\left(\;e_{j}^{\bar{q}}-{\bar{e}}_{j}^{\bar{q}}\;\right)\;= \end{array}$$

(A60b) $$\begin{array}{c} =\;\sum_{j}\left[\;{\bar{\delta }}_{0}\;\delta_{ij}\;-\;\sum_{k}\varepsilon_{ikj}\;{\bar{\delta }}_{k}\;\right]\left(\;e_{j}^{\bar{q}}-{\bar{e}}_{j}^{\bar{q}}\;\right), \end{array}$$

being $\delta_{ij}$ the Kronecker delta. Returning to matrix notation, the linear approximation of $N\left(e^{\bar{q}}\right)$ is

(A61) $$N\left(\;e^{\bar{q}}\;\right)\;=\;\left[\;{\bar{\delta }}_{0}\;I-{\left[\;\bar{\delta }\;\right]}_{\times }\;\right]\left(e^{\bar{q}}-{\bar{e}}^{\bar{q}}\right)\;+\;O\left(∥e^{\bar{q}}-{\bar{e}}^{\bar{q}}{∥}^{2}\right).$$

On the other hand, evaluating (A57) at ${\bar{e}}^{\bar{q}}$ we obtain the zeroth order approximation:

(A62a) $$\begin{array}{cc} D\left(\;{\bar{e}}^{\bar{q}}\;\right)\; & =\;2\;{\bar{\delta }}_{0}+\bar{\delta }\cdot 2\;\frac{\bar{\delta }}{{\bar{\delta }}_{0}}\;+\;O\left(∥e^{\bar{q}}-{\bar{e}}^{\bar{q}}∥\right)\;= \end{array}$$

(A62b) $$\begin{array}{c} =\;\frac{2}{{\bar{\delta }}_{0}}\;\underset{1}{\underset{︸}{\left({\bar{\delta }}_{0}^{2}\;+\;{∥\bar{\delta }∥}^{2}\right)}}\;+\;O\left(∥e^{\bar{q}}-{\bar{e}}^{\bar{q}}∥\right)\;= \end{array}$$

(A62c) $$\begin{array}{c} =\;\frac{2}{{\bar{\delta }}_{0}}\;+\;O\left(∥e^{\bar{q}}-{\bar{e}}^{\bar{q}}∥\right). \end{array}$$

Finally, combining (A61) and (A62c) we can compute the linear approximation of (A6):

(A63a) $$\begin{array}{cc} e^{\bar{p}}\left(\;e^{\bar{q}}\;\right)\; & =\;2\;\left\{\left[\;{\bar{\delta }}_{0}\;I\;-\;{\left[\;\bar{\delta }\;\right]}_{\times }\;\right]\left(e^{\bar{q}}-{\bar{e}}^{\bar{q}}\right)\;+\;O\left(∥e^{\bar{q}}-{\bar{e}}^{\bar{q}}{∥}^{2}\right)\right\}\left[\;\frac{{\bar{\delta }}_{0}}{2}\;+\;O\left(∥e^{\bar{q}}-{\bar{e}}^{\bar{q}}∥\right)\;\right]\;= \end{array}$$

(A63b) $$\begin{array}{c} =\;{\bar{\delta }}_{0}\;\left[\;{\bar{\delta }}_{0}\;I\;-\;{\left[\;\bar{\delta }\;\right]}_{\times }\;\right]\left(e^{\bar{q}}-{\bar{e}}^{\bar{q}}\right)\;+\;O\left(∥e^{\bar{q}}-{\bar{e}}^{\bar{q}}{∥}^{2}\right). \end{array}$$

### Appendix C.3. Modified Rodrigues Parameters

First, let us denote the numerator of (A9) as $N(e^{\bar{q}})$, and its denominator as $D(e^{\bar{q}})$:

(A64) $$\begin{array}{cc} N\left(\;e^{\bar{q}}\;\right)\; & =\;8\;{\bar{\delta }}_{0}\;e^{\bar{q}}\;-\;\left(16\;-\;∥e^{\bar{q}}{∥}^{2}\right)\;\bar{\delta }\;-\;8\;\bar{\delta }\times e^{\bar{q}}, \end{array}$$

(A65) $$\begin{array}{cc} D\left(\;e^{\bar{q}}\;\right)\; & =\;16+∥e^{\bar{q}}{∥}^{2}+{\bar{\delta }}_{0}\;\left(16\;-\;∥e^{\bar{q}}{∥}^{2}\right)+8\;\bar{\delta }\cdot e^{\bar{q}}. \end{array}$$

Now let us evaluate (A64) at ${\bar{e}}^{\bar{q}}$:

(A66a) Ne¯q¯=8δ¯0e¯q¯︷4δ¯/(1+δ¯0)−16−∥e¯q¯∥2︷16∥δ¯∥2/(1+δ¯0)2δ¯−8δ¯×e¯q¯︷0(e¯q¯∥δ¯)=0(e¯q¯∥δ¯)=

(A66b) $$\begin{array}{c} =\;\frac{\;16\;\bar{\delta }\;}{\;1+{\bar{\delta }}_{0}\;}\;\left[\;2\;{\bar{\delta }}_{0}\;-\;\left(1\;+\;{\bar{\delta }}_{0}\;-\;\frac{\;\overset{1-{\bar{\delta }}_{0}^{2}}{\overset{︷}{∥\bar{\delta }{∥}^{2}}}\;}{\;1+{\bar{\delta }}_{0}\;}\right)\right]\;=\;0. \end{array}$$

Then, as with the RP chart, the approximation of $N\left(e^{\bar{q}}\right)$ does not have terms of order O(1), and we will only need to approximate $D\left(e^{\bar{q}}\right)$ to the zeroth order.

We can write (A64) as

(A67) $$N_{i}\left(\;e^{\bar{q}}\;\right)\;=\;8\;{\bar{\delta }}_{0}\;e_{i}^{\bar{q}}\;-\;\left(16-\sum_{k}{\left(e_{k}^{\bar{q}}\right)}^{2}\right)\;{\bar{\delta }}_{i}\;-\;8\sum_{lm}\varepsilon_{ilm}\;{\bar{\delta }}_{l}\;e_{m}^{\bar{q}},$$

with $\varepsilon_{ilm}$ the Levi-Civita symbol. Applying (44) to (A67),

(A68a) $$\begin{array}{cc} N_{i}\left(\;e^{\bar{q}}\;\right)\; & \approx \;\sum_{j}{\left[\;8\;{\bar{\delta }}_{0}\;\delta_{ij}\;+\;\sum_{k}2\;e_{k}^{\bar{q}}\;\delta_{kj}\;{\bar{\delta }}_{i}\;-\;8\sum_{lm}\varepsilon_{ilm}\;{\bar{\delta }}_{l}\;\delta_{mj}\;\right]}_{e^{\bar{q}}={\bar{e}}^{\bar{q}}}\left(\;e_{j}^{\bar{q}}-{\bar{e}}_{j}^{\bar{q}}\;\right)\;= \end{array}$$

(A68b) $$\begin{array}{c} =\;\sum_{j}\left[\;8\;{\bar{\delta }}_{0}\;\delta_{ij}\;+\;2\;{\bar{e}}_{j}^{\bar{q}}\;{\bar{\delta }}_{i}\;-\;8\sum_{l}\varepsilon_{ilj}\;{\bar{\delta }}_{l}\;\right]\left(\;e_{j}^{\bar{q}}-{\bar{e}}_{j}^{\bar{q}}\;\right), \end{array}$$

being $\delta_{ij}$ the Kronecker delta. Returning to matrix notation, the linear approximation of $N\left(\;e^{\bar{q}}\;\right)$ is

(A69a) $$\begin{array}{cc} N\left(\;e^{\bar{q}}\;\right)\; & =\;\left[\;8\;{\bar{\delta }}_{0}\;I\;+\;2\;\bar{\delta }\;{\left({\bar{e}}^{\bar{q}}\right)}^{T}\;-\;8\;{\left[\;\bar{\delta }\;\right]}_{\times }\;\right]\left(e^{\bar{q}}-{\bar{e}}^{\bar{q}}\right)\;+\;O\left(∥e^{\bar{q}}-{\bar{e}}^{\bar{q}}{∥}^{2}\right)\;= \end{array}$$

(A69b) $$\begin{array}{c} =\;8\;\left[\;{\bar{\delta }}_{0}\;I\;+\;\frac{\;\bar{\delta }\;{\bar{\delta }}^{T}\;}{\;1+{\bar{\delta }}_{0}\;}\;-\;{\left[\;\bar{\delta }\;\right]}_{\times }\;\right]\left(e^{\bar{q}}-{\bar{e}}^{\bar{q}}\right)\;+\;O\left(∥e^{\bar{q}}-{\bar{e}}^{\bar{q}}{∥}^{2}\right). \end{array}$$

On the other hand, evaluating (A65) at ${\bar{e}}^{\bar{q}}$ we obtain the zeroth order approximation:

(A70a) $$\begin{array}{cc} D\left(\;{\bar{e}}^{\bar{q}}\;\right)\; & \approx \;16+16\;\frac{\;∥\bar{\delta }{∥}^{2}\;}{\;{\left(1+{\bar{\delta }}_{0}\right)}^{2}\;}+{\bar{\delta }}_{0}\;\left(16\;-\;16\;\frac{\;∥\bar{\delta }{∥}^{2}\;}{\;{\left(1+{\bar{\delta }}_{0}\right)}^{2}\;}\right)+8\;\bar{\delta }\cdot 4\;\frac{\;\bar{\delta }\;}{\;1+{\bar{\delta }}_{0}\;}\;= \end{array}$$

(A70b) $$\begin{array}{c} =\;\frac{\;16\;}{\;1+{\bar{\delta }}_{0}\;}\;\left[\;\left(1+{\bar{\delta }}_{0}\right)+\frac{\;∥\bar{\delta }{∥}^{2}\;}{\;1+{\bar{\delta }}_{0}\;}+{\bar{\delta }}_{0}\left(\;\left(1+{\bar{\delta }}_{0}\right)\;-\;\frac{\;∥\bar{\delta }{∥}^{2}\;}{\;1+{\bar{\delta }}_{0}\;}\;\right)+2\;{∥\bar{\delta }∥}^{2}\;\right]\;= \end{array}$$

(A70c) $$\begin{array}{c} =\;\frac{\;16\;}{\;1+{\bar{\delta }}_{0}\;}\;\left[\;2+{\bar{\delta }}_{0}\left(\;2\;{\bar{\delta }}_{0}\;\right)+2\;\left(1-{\bar{\delta }}_{0}^{2}\right)\;\right]\;=\;\frac{\;64\;}{\;1+{\bar{\delta }}_{0}\;}, \end{array}$$

where we have used the equality $∥\bar{\delta }{∥}^{2}=1-{\bar{\delta }}_{0}^{2}$ for unit quaternions. Finally, combining (A69b) and (A70c) we can compute the linear approximation of (A9):

(A71a) $$\begin{array}{cc} e^{\bar{p}}\left(\;e^{\bar{q}}\;\right)\; & =\;4\;\left\{8\;\left[\;{\bar{\delta }}_{0}\;I\;+\;\frac{\;\bar{\delta }\;{\bar{\delta }}^{T}\;}{\;1+{\bar{\delta }}_{0}\;}\;-\;{\left[\;\bar{\delta }\;\right]}_{\times }\;\right]\left(e^{\bar{q}}-{\bar{e}}^{\bar{q}}\right)\;+\;O\left(∥e^{\bar{q}}-{\bar{e}}^{\bar{q}}{∥}^{2}\right)\right\}\left[\;\frac{\;1+{\bar{\delta }}_{0}\;}{\;64\;}\;+\;O\left(∥e^{\bar{q}}-{\bar{e}}^{\bar{q}}∥\right)\;\right]\;= \end{array}$$

(A71b) $$\begin{array}{c} =\;\frac{\;1+{\bar{\delta }}_{0}\;}{\;2\;}\;\left[\;{\bar{\delta }}_{0}\;I\;+\;\frac{\;\bar{\delta }\;{\bar{\delta }}^{T}\;}{\;1+{\bar{\delta }}_{0}\;}\;-\;{\left[\;\bar{\delta }\;\right]}_{\times }\;\right]\left(e^{\bar{q}}-{\bar{e}}^{\bar{q}}\right)\;+\;O\left(∥e^{\bar{q}}-{\bar{e}}^{\bar{q}}{∥}^{2}\right)\;= \end{array}$$

(A71c) $$\begin{array}{c} =\;\frac{1}{2}\left[\;\left(1+{\bar{\delta }}_{0}\right)\left({\bar{\delta }}_{0}\;I\;-\;{\left[\;\bar{\delta }\;\right]}_{\times }\right)+\bar{\delta }\;{\bar{\delta }}^{T}\;\right]\left(e^{\bar{q}}-{\bar{e}}^{\bar{q}}\right)\;+\;O\left(∥e^{\bar{q}}-{\bar{e}}^{\bar{q}}{∥}^{2}\right). \end{array}$$

### Appendix C.4. Rotation Vector

Let us start evaluating the vector $\delta^{\bar{p}}$ in (A12) and (A13) at the point ${\bar{e}}^{\bar{q}}$:

(A72) δp¯e¯q¯=δ¯0e¯^q¯sin∥e¯q¯∥2︸δ¯−cos∥e¯q¯∥2︸δ¯0δ¯︷0=0−δ¯×e¯^q¯sin∥e¯q¯∥2︸δ¯︷0=0=0.

Then, the first order approximation of $\delta^{\bar{p}}$ around ${\bar{e}}^{\bar{q}}$ will have the form

(A73) $$\delta^{\bar{p}}\;=\;\tilde{T}\;\left(\;e^{\bar{q}}\;-\;{\bar{e}}^{\bar{q}}\;\right)\;+\;O\left(∥e^{\bar{q}}\;-\;{\bar{e}}^{\bar{q}}{∥}^{2}\right),$$

and $∥\delta^{\bar{p}}∥\to 0$ as $e^{\bar{q}}\to {\bar{e}}^{\bar{q}}$. Taking the Taylor series of the arcsinx,

(A74) $$\frac{\;\arcsin∥\delta^{\bar{p}}∥\;}{\;∥\delta^{\bar{p}}∥\;}\;=\;\frac{\;∥\delta^{\bar{p}}∥+O\left(∥\delta^{\bar{p}}{∥}^{3}\right)\;}{\;∥\delta^{\bar{p}}∥\;}\;=\;1\;+\;O\left(∥\delta^{\bar{p}}{∥}^{2}\right)\;=\;1\;+\;O\left(∥e^{\bar{q}}-{\bar{e}}^{\bar{q}}{∥}^{2}\right),$$

so that (A12) is linearized as

(A75) $$2\;\frac{\;\delta^{\bar{p}}\;}{\;∥\delta^{\bar{p}}∥\;}\arcsin∥\delta^{\bar{p}}∥\;=\;2\;\tilde{T}\;\left(\;e^{\bar{q}}\;-\;{\bar{e}}^{\bar{q}}\;\right)\;+\;O\left(∥e^{\bar{q}}\;-\;{\bar{e}}^{\bar{q}}{∥}^{2}\right).$$

We only lack the $\tilde{T}$ matrix. We will need the linear approximations of $\cos\left(∥e^{\bar{q}}∥/2\right)$ and ${\hat{e}}^{\bar{q}}\;\sin\left(∥e^{\bar{q}}∥/2\right)$ around ${\bar{e}}^{\bar{q}}$. To this end we will first obtain the linear approximation of ∥x∥:

(A76a) $$\begin{array}{cc} ∥x∥\; & =\;\sqrt{\sum_{k}x_{k}^{2}\;}\;= \end{array}$$

(A76b) $$\begin{array}{c} =\;∥\bar{x}∥\;+\;\sum_{j}{\left[\frac{\;\sum_{k}x_{k}\;\delta_{kj}\;}{\;\sqrt{\sum_{k}x_{k}^{2}\;}\;}\right]}_{x=\bar{x}}\;\;\left(x_{j}-{\bar{x}}_{j}\right)\;+\;O\left(∥x-\bar{x}{∥}^{2}\right)\;= \end{array}$$

(A76c) $$\begin{array}{c} =\;∥\bar{x}∥\;+\;\sum_{j}\left[\frac{\;{\bar{x}}_{j}\;}{\;\sqrt{\sum_{k}{\bar{x}}_{k}^{2}\;}\;}\right]\left(x_{j}-{\bar{x}}_{j}\right)\;+\;O\left(∥x-\bar{x}{∥}^{2}\right)\;= \end{array}$$

(A76d) $$\begin{array}{c} =\;∥\bar{x}∥\;+\;{\hat{\bar{x}}}^{T}\left(x-\bar{x}\right)\;+\;O\left(∥x-\bar{x}{∥}^{2}\right). \end{array}$$

Noticing that

(A77) $$\frac{\;\partial \;∥x∥\;}{\;\partial x\;}\;=\;{\hat{\bar{x}}}^{T}+O\left(∥x-\bar{x}∥\right),$$

our computations are straightforward:

(A78a) $$\begin{array}{cc} \cos\left(\frac{\;∥x∥\;}{\;2\;}\right)\; & =\;\cos\left(\frac{\;∥\bar{x}∥\;}{2}\right)\;-{\left[\sin\left(\frac{\;∥x∥\;}{\;2\;}\right)\frac{1}{2}\left[\;{\hat{\bar{x}}}^{T}+O\left(∥x-\bar{x}∥\right)\right]\right]}_{x=\bar{x}}\;\;\left(x-\bar{x}\right)\;+\;O\left(∥x-\bar{x}{∥}^{2}\right)\;= \end{array}$$

(A78b) $$\begin{array}{c} =\;\cos\left(\frac{\;∥\bar{x}∥\;}{2}\right)\;-\;\frac{1}{2}\sin\left(\frac{\;∥\bar{x}∥\;}{\;2\;}\right){\hat{\bar{x}}}^{T}\left(x-\bar{x}\right)\;+\;O\left(∥x-\bar{x}{∥}^{2}\right). \end{array}$$

For our particular case,

(A79) $$\cos\left(\frac{\;∥e^{\bar{q}}∥\;}{\;2\;}\right)\;=\;{\bar{\delta }}_{0}\;-\;\frac{1}{2}\;{\bar{\delta }}^{T}\left(e^{\bar{q}}-{\bar{e}}^{\bar{q}}\right)\;+\;O\left(∥e^{\bar{q}}-{\bar{e}}^{\bar{q}}{∥}^{2}\right).$$

On the other hand,

(A80a) $$\begin{array}{c} \frac{\;\sin\left(\frac{\;∥x∥\;}{\;2\;}\right)\;}{\;∥x∥\;}\;= \end{array}$$

(A80b) $$\begin{array}{c} =\;\frac{\;\sin\left(\frac{\;∥\bar{x}∥\;}{\;2\;}\right)\;}{\;∥\bar{x}∥\;}\;+\;{\left[\left(\frac{\;\cos\left(\frac{\;∥x∥\;}{\;2\;}\right)\;}{\;∥x∥\;}\;\frac{1}{2}\;-\;\frac{\;\sin\left(\frac{\;∥x∥\;}{\;2\;}\right)\;}{{\;∥x∥}^{2}\;}\right)\left[\;{\hat{\bar{x}}}^{T}+O\left(∥x-\bar{x}∥\right)\right]\right]}_{x=\bar{x}}\;\;\left(x-\bar{x}\right)\;+\;O\left(∥x-\bar{x}{∥}^{2}\right)\;= \end{array}$$

(A80c) $$\begin{array}{c} =\;\frac{\;\sin\left(\frac{\;∥\bar{x}∥\;}{\;2\;}\right)\;}{\;∥\bar{x}∥\;}\;+\;\left[\left(\frac{\;\cos\left(\frac{\;∥\bar{x}∥\;}{\;2\;}\right)\;}{\;∥\bar{x}∥\;}\;\frac{1}{2}\;-\;\frac{\;\sin\left(\frac{\;∥\bar{x}∥\;}{\;2\;}\right)\;}{\;∥\bar{x}{∥}^{2}\;}\right){\hat{\bar{x}}}^{T}\right]\left(x-\bar{x}\right)\;+\;O\left(∥x-\bar{x}{∥}^{2}\right). \end{array}$$

Now, taking $x=\bar{x}+\left(x-\bar{x}\right)$ we arrive at

(A81a) $$\begin{array}{c} \frac{\;x\;}{\;∥x∥\;}\;\sin\left(\frac{\;∥x∥\;}{\;2\;}\right)\;=\;\left[\bar{x}+\left(x-\bar{x}\right)\right]\frac{\;\sin\left(\frac{\;∥x∥\;}{\;2\;}\right)\;}{\;∥x∥\;}\;= \end{array}$$

(A81b) $$\begin{array}{c} =\;\hat{\bar{x}}\sin\left(\frac{\;∥\bar{x}∥\;}{\;2\;}\right)\;+\;\left[\frac{\;\sin\left(\frac{\;∥\bar{x}∥\;}{\;2\;}\right)\;}{\;∥\bar{x}∥\;}\;+\;\frac{1}{2}\cos\left(\frac{\;∥\bar{x}∥\;}{\;2\;}\right)\hat{\bar{x}}\;{\hat{\bar{x}}}^{T}-\frac{\;\sin\left(\frac{\;∥\bar{x}∥\;}{\;2\;}\right)\;}{\;∥\bar{x}∥\;}\hat{\bar{x}}\;{\hat{\bar{x}}}^{T}\right]\left(x-\bar{x}\right)\;+\;O\left(∥x-\bar{x}{∥}^{2}\right). \end{array}$$

For our particular case,

(A82a) $$\begin{array}{cc} \frac{\;e^{\bar{q}}\;}{\;∥e^{\bar{q}}∥\;}\;\sin\left(\frac{\;∥e^{\bar{q}}∥\;}{\;2\;}\right)\; & =\;\bar{\delta }\;+\;\left[\frac{\;∥\bar{\delta }∥\;}{\;2\;\arcsin∥\bar{\delta }∥\;}\;+\;\frac{\;{\bar{\delta }}_{0}\;}{\;2\;}\;\hat{\bar{\delta }}\;{\hat{\bar{\delta }}}^{T}\;-\;\frac{\;∥\bar{\delta }∥\;}{\;2\;\arcsin∥\bar{\delta }∥\;}\;\hat{\bar{\delta }}\;{\hat{\bar{\delta }}}^{T}\right]\left(e^{\bar{q}}-{\bar{e}}^{\bar{q}}\right)\;+\;O\left(∥e^{\bar{q}}-{\bar{e}}^{\bar{q}}{∥}^{2}\right)\;= \end{array}$$

(A82b) $$\begin{array}{c} =\;\bar{\delta }\;+\;\frac{1}{2}\left[\left(I-\hat{\bar{\delta }}\;{\hat{\bar{\delta }}}^{T}\right)\frac{\;∥\bar{\delta }∥\;}{\;\arcsin∥\bar{\delta }∥\;}\;+\;{\bar{\delta }}_{0}\;\hat{\bar{\delta }}\;{\hat{\bar{\delta }}}^{T}\right]\left(e^{\bar{q}}-{\bar{e}}^{\bar{q}}\right)\;+\;O\left(∥e^{\bar{q}}-{\bar{e}}^{\bar{q}}{∥}^{2}\right). \end{array}$$

Finally, we just have to replace (A79) and (A82b) in (A13) to obtain the required linear approximation. Returning to the original notation we have

(A83a) $$\begin{array}{c} 2\;\frac{\;\delta^{\bar{p}}\;}{\;∥\delta^{\bar{p}}∥\;}\arcsin∥\delta^{\bar{p}}∥\;=\;2\;\delta^{\bar{p}}\;+\;O\left(∥e^{\bar{q}}-{\bar{e}}^{\bar{q}}{∥}^{2}\right)\;= \end{array}$$

(A83b) $$\begin{array}{c} =\;2\;{\bar{\delta }}_{0}\;{\hat{e}}^{\bar{q}}\;\sin\left(\frac{∥e^{\bar{q}}∥}{2}\right)\;-\;2\;\cos\left(\frac{∥e^{\bar{q}}∥}{2}\right)\;\bar{\delta }\;-\;2\;\bar{\delta }\times {\hat{e}}^{\bar{q}}\;\sin\left(\frac{∥e^{\bar{q}}∥}{2}\right)\;+\;O\left(∥e^{\bar{q}}-{\bar{e}}^{\bar{q}}{∥}^{2}\right)\;=\\ =\;2\;{\bar{\delta }}_{0}\;\bar{\delta }\;+\;{\bar{\delta }}_{0}\left[\;\left(I\;-\;\hat{\bar{\delta }}\;{\hat{\bar{\delta }}}^{T}\right)\frac{\;∥\bar{\delta }∥\;}{\;\arcsin∥\bar{\delta }∥\;}+{\bar{\delta }}_{0}\;\hat{\bar{\delta }}\;{\hat{\bar{\delta }}}^{T}\right]\left(e^{\bar{q}}-{\bar{e}}^{\bar{q}}\right)\;-\;\left(2\;{\bar{\delta }}_{0}\;-\;{\bar{\delta }}^{T}\left(e^{\bar{q}}-{\bar{e}}^{\bar{q}}\right)\right)\bar{\delta }\;+ \end{array}$$

(A83c) $$\begin{array}{c} \;-\;\bar{\delta }\times \left\{\;2\;\bar{\delta }+\left[\left(I\;-\;\hat{\bar{\delta }}\;{\hat{\bar{\delta }}}^{T}\right)\frac{\;∥\bar{\delta }∥\;}{\;\arcsin∥\bar{\delta }∥\;}+{\bar{\delta }}_{0}\;\hat{\bar{\delta }}\;{\hat{\bar{\delta }}}^{T}\right]\left(e^{\bar{q}}-{\bar{e}}^{\bar{q}}\right)\;\right\}\;+\;O\left(∥e^{\bar{q}}-{\bar{e}}^{\bar{q}}{∥}^{2}\right)\;= \end{array}$$

(A83d) $$\begin{array}{c} =\;\left[\left({\bar{\delta }}_{0}\;\left(I\;-\;\hat{\bar{\delta }}\;{\hat{\bar{\delta }}}^{T}\right)\;-\;{\left[\;\bar{\delta }\;\right]}_{\times }\right)\frac{\;∥\bar{\delta }∥\;}{\;\arcsin∥\bar{\delta }∥\;}+{\bar{\delta }}_{0}^{2}\;\hat{\bar{\delta }}\;{\hat{\bar{\delta }}}^{T}+\bar{\delta }\;{\bar{\delta }}^{T}\right]\left(e^{\bar{q}}-{\bar{e}}^{\bar{q}}\right)\;+\;O\left(∥e^{\bar{q}}-{\bar{e}}^{\bar{q}}{∥}^{2}\right)\;= \end{array}$$

(A83e) $$\begin{array}{c} =\;\left[\;\left({\bar{\delta }}_{0}\;\left(I\;-\;\hat{\bar{\delta }}\;{\hat{\bar{\delta }}}^{T}\right)\;-\;{\left[\;\bar{\delta }\;\right]}_{\times }\right)\frac{\;∥\bar{\delta }∥\;}{\;\arcsin∥\bar{\delta }∥\;}+\hat{\bar{\delta }}\;{\hat{\bar{\delta }}}^{T}\;\right]\left(e^{\bar{q}}-{\bar{e}}^{\bar{q}}\right)\;+\;O\left(∥e^{\bar{q}}-{\bar{e}}^{\bar{q}}{∥}^{2}\right). \end{array}$$

Note that the linear approximations of our transition maps are valid for $e^{\bar{q}}$ near of ${\bar{e}}^{\bar{q}}$. However, we have not made any assumption about the $\bar{\delta }$ quaternion. This means that our linear approximations are exact for any $\bar{\delta }=\varphi^{-1}\left({\bar{e}}^{\bar{q}}\right)$ in the domain of each T-matrix, provided that $e^{\bar{q}}$ is close enough to ${\bar{e}}^{\bar{q}}$.
